# Mixoplankton interferences in dilution grazing experiments

**DOI:** 10.1038/s41598-021-03176-0

**Published:** 2021-12-13

**Authors:** Guilherme Duarte Ferreira, Filomena Romano, Nikola Medić, Paraskevi Pitta, Per Juel Hansen, Kevin J. Flynn, Aditee Mitra, Albert Calbet

**Affiliations:** 1 Institut de Ciències del Mar, CSIC, Pg. Marítim de la Barceloneta, 37-49, 08003 Barcelona, Spain; 2 Marine Biological Section, University of Copenhagen, 3000 Helsingør, Denmark; 3 Institute of Oceanography, Hellenic Centre for Marine Research, PO Box 2214, 71003 Heraklion, Greece; 4 Plymouth Marine Laboratory, Prospect Place, Plymouth, PL1 3DH UK; 5 School of Earth and Environmental Sciences, Cardiff University, Park Place, Cardiff, CF10 3AT UK

**Keywords:** Biological techniques, Ecology

## Abstract

It remains unclear as to how mixoplankton (coupled phototrophy and phagotrophy in one cell) affects the estimation of grazing rates obtained from the widely used dilution grazing technique. To address this issue, we prepared laboratory-controlled dilution experiments with known mixtures of phyto-, protozoo-, and mixoplankton, operated under different light regimes and species combinations. Our results evidenced that chlorophyll is an inadequate proxy for phytoplankton when mixoplankton are present. Conversely, species-specific cellular counts could assist (although not fully solve) in the integration of mixoplanktonic activity in a dilution experiment. Moreover, cell counts can expose prey selectivity patterns and intraguild interactions among grazers. Our results also demonstrated that whole community approaches mimic reality better than single-species laboratory experiments. We also confirmed that light is required for protozoo- and mixoplankton to correctly express their feeding activity, and that overall diurnal grazing is higher than nocturnal. Thus, we recommend that a detailed examination of initial and final plankton communities should become routine in dilution experiments, and that incubations should preferably be started at the beginning of both day and night periods. Finally, we hypothesize that in silico approaches may help disentangle the contribution of mixoplankton to the community grazing of a given system.

## Introduction

The dilution grazing technique^1^ is the most widely used method to measure microplankton grazing in the field, with more than one hundred studies on the topic throughout the world^2^. It provides the rates of “phytoplankton” growth and “microzooplankton” grazing with a relatively simple experimental design. The rationale behind the method comes from the decrease in the encounter rates between predators and their prey as the whole community is diluted. Additionally, it assumes that phytoplankton growth is affected neither by the dilution factor nor by the presence of other phytoplankton species/individuals^2^.

The technique, as any other, is, however, beset by variouslimitations problems, which have been extensively discussed in several papers (see^2–4^, and references therein). A particular challenge, and one that is often neglected, is the consequences of the presence of mixoplankton in the incubations^2,5^. Mixoplankton are protists that combine photo-autotrophy, osmo-heterotrophy, and phago-heterotrophy^6^; organisms that combine the former two modes of nutrient acquisition are termed phytoplankton whereas combining the latter two results in protozooplankton^6^. Mixoplankton can be divided according to their acquisition of chloroplasts into two major functional groups, the Constitutive and the Non-Constitutive mixoplankton^6,7^). The former possess their own chloroplasts, while the latter have to retain them from ingested photosynthetic prey.

In the original description of the dilution technique^1^, the growth of the “phytoplankton” prey was assessed by using chlorophyll *a* (Chl *a*) as a proxy for its biomass, and grazing was assumed to be exclusively due to predatory activity of “microzooplankton” (i.e., de facto protozooplankton). Classic methods for estimating primary or secondary productivity do not recognise the complexity of involving mixoplankton growth^8^, and the numerous approaches to quantify grazing fail to distinguish mixoplanktonic and protozooplanktonic activities^6,9^. Thus, the presence of mixoplankton is obviously integrated in a traditional dilution setting. However, the simultaneous display of “phytoplankton-like” phototrophic and “microzooplankton-like” phagotrophic activities means that it is, currently, impossible to discriminate mixoplanktonic and protozooplanktonic contributions to grazing^5,10,11^.

The presence of mixoplankton during the dilution incubations would not represent a serious shortcoming if mixoplankton were seldom present in the studied water. However, mixoplankton are not only ubiquitous^12,13^, but also phylogenetically diverse, and can be found across a wide size spectrum^6^. Therefore, mixoplankton are expected to be very important grazers in marine systems, and even dominant in some^8^. Nonetheless, the studies that quantify their grazing impact in situ are not very common (e.g., for bacterivory^14,15^; for herbivory^5,16,17^) due to methodological difficulties^9^.

Another possible issue with the dilution grazing technique is the incongruence between grazing rates derived from the technique and those obtained in the laboratory with single-species predator–prey experiments^18^. In the laboratory, the experimental determination of feeding rates typically involves the direct measurement of prey and predator abundances over a given period^19^. In the field, however, the complexity of the system poses a significant challenge for the accurate estimation of response function parameters for microzooplankton^20^ as these cannot be directly measured^1,3^. This discrepancy is not surprising because of the multitude of biological interactions that take place within a given water column, which can (and likely will) alter individual and community grazing rates. Some of these major biological factors include the production of allelopathic compounds (e.g.,^21,22^), intraguild predation and trophic cascades (e.g.,^3,11,23–25^), and prey selectivity (e.g.,^23,26–28^). Given the omnipresent nature of these features in marine ecosystems, it becomes clear that they cannot be ignored when interpreting dilution grazing experiments. The presence of mixoplankton, for the above-mentioned reasons, further complicates the situation.

With these matters in mind, we conducted several dilution grazing experiments in the laboratory, with artificial food webs created from known mixtures of phyto-, protozoo- and mixoplankton species. Additionally, we prepared control treatments (that cannot be included in field experiments) containing only prey, and combinations of a single predator with the prey, to explore individual dynamics during the incubation. Thus, our primary aim was to understand some of the underlying forces of a dilution experiment containing mixoplanktonic grazers, by altering some factors known to modulate grazing like irradiance and prey competition. Therefore, the experiments were conducted under regular diel light cycle conditions and also in complete darkness because light can act both as a resource for phototrophic growth and as a modulating factor for grazing^29,30^. Dark incubations could serve to provide information on the contribution of mixoplanktonic activity into dilution grazing experiments. These additional experiments, as controlled scenarios, provide added information for interpreting a dilution grazing experiment and could, ultimately, be used for in silico simulations of dilution grazing experiments.

## Results

### Dilution grazing experiments

**Figure 1:**
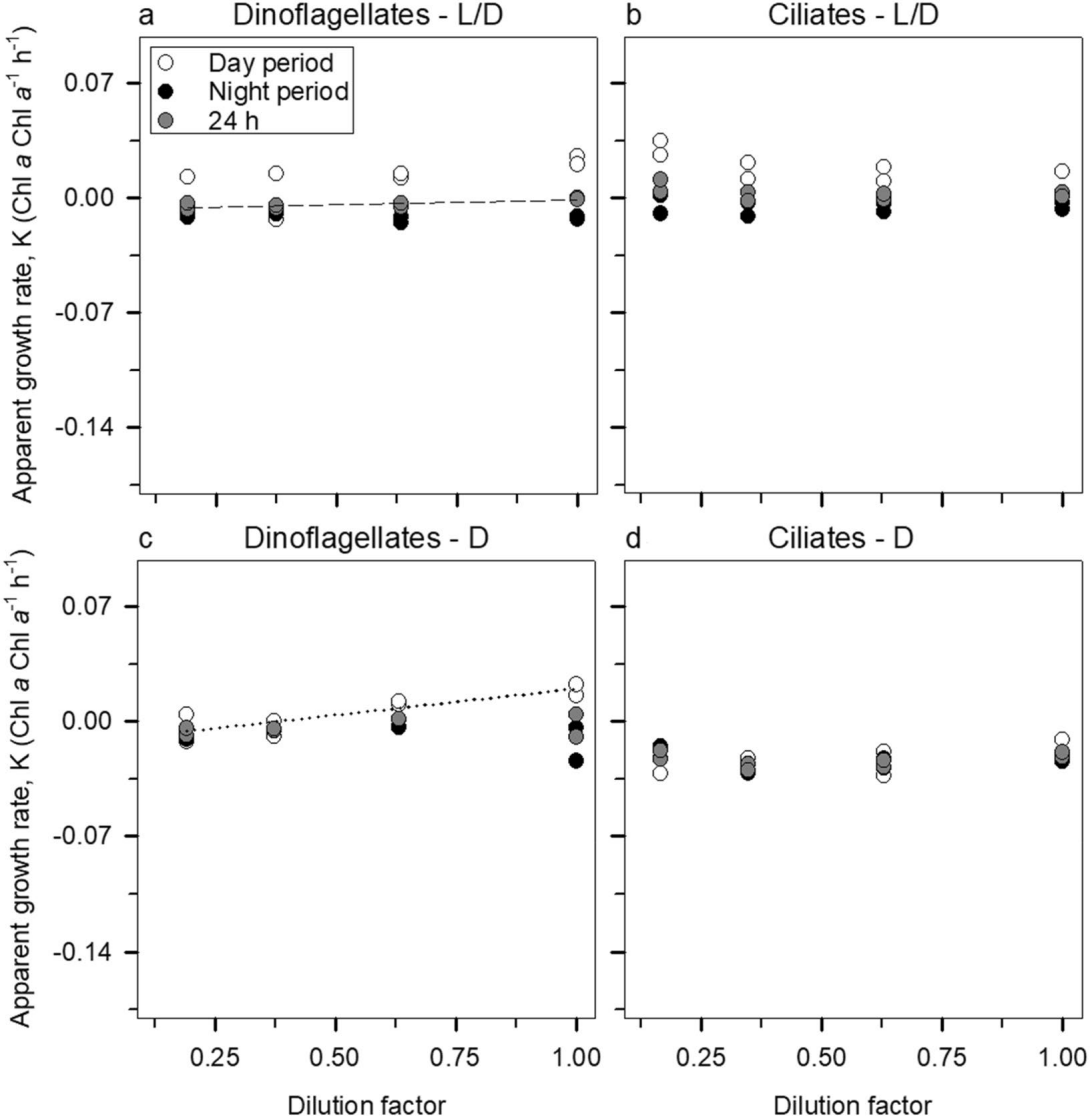
Chl *a*-based dilution grazing experiment results. The left panels (**a** and **c**) show experiments with dinoflagellates and the right panels (**b** and **d**) correspond to experiments with ciliates. The top section is relative to the L/D treatment whereas the bottom one relates to D treatment. Plotted regressions imply a significant slope (*P* < 0.05). Dotted regression lines correspond to the day period and dashed lines to the integrated 24 h incubations.

The majority of the dilution grazing incubations yielded non-significant grazing rates (*P* > 0.05) when based on Chl *a* (Fig. 1). The only exceptions were the experiment with dinoflagellates (Fig. 1a, c), but the slopes of the regressions were positive on both instances (Table 1). In the occasions where mixoplankton represent a relevant shear of the pigmented community, it is thus challenging to determine the actual grazing mortality using the traditional dilution approach of tracking only Chl *a*..

**Table 1:** Summary of growth (µ, h^−l^) and grazing (g, h^−l^) rates calculated from the slopes of dilution grazing experiments at the different periods of the day.

Exp	Rate, Species	Day	R^2^	Day	R^2^	Night	R^2^	Night	R^2^	24 h	R^2^	24 h	R^2^
Dinoflagellates	µ, Total Chl *a*	− 0.0059	0.47	**− 0.0121**	**0.74**^**++**^	− 0.0081	0.31	**− 0.0032**	**0.13**	− 0.0071	0.60^+^	**− 0.0061**	**0.12**
	g, Total Chl *a*	− 0.0285		**− 0.0321**		0.0049		**0.0079**		− 0.0059		**− 0.0053**	
	µ, *R. salina*	− 0.0431	0.77^+^	**0.0065**	**0.03**	− 0.0132	0.80^+^	**− 0.0102**	**0.06**	− 0.0229	0.85^++^	**− 0.0039**	**0.03**
	g, *R. salina*	0.1091^#^		**0.0102**		0.0311^#^		**− 0.0025**		0.0575^#^		**0.0025**	
	µ, *C. weissflogii*	0.0280	0.15	**− 0.0107**	**0.21**	0.0249	0.19	**− 0.0172**	**0.76**^**++**^	0.0259	0.50^+^	**− 0.0150**	**0.79**^**++**^
	g, *C. weissflogii*	− 0.0126		**− 0.0167**		− 0.0089		**− 0.0296**		− 0.0104		**− 0.0253**	
	µ, *K. armiger*	− 0.0278	0.48	**0.0022**	**0.03**	0.0110	0.23	**− 0.0049**	**0.81**^**++**^	− 0.0019	0.09	**− 0.0024**	**0.87**^**++**^
	g, *K. armiger*	− 0.0311		**0.0073**		0.0096		**0.0431**		− 0.0039		**0.0312**	
	µ, *G. dominans*	− 0.0043	0.49	**0.0285**	**0.00**	0.0249	0.33	**− 0.0004**	**0.01**	0.0152	0.01	**0.0093**	**0.00**
	g, *G. dominans*	− 0.0377		**− 0.0019**		0.0147		**0.0028**		− 0.0027		**0.0012**	
Ciliates	µ, Total Chl *a*	0.0277	0.36	**− 0.0281**	**0.13**	− 0.0056	0.00	**− 0.0233**	**0.00**	0.0052	0.16	**− 0.0248**	**0.03**
	g, Total Chl *a*	0.0171		**− 0.0087**		0.0001		**0.0010**		0.0047		**− 0.0022**	
	µ, *R. salina*	− 0.0241	0.87^++^	**− 0.0432**	**0.92**^**++**^	− 0.0081	0.36	**− 0.0075**	**0.01**	− 0.0134	0.42	**− 0.0247**	**0.51**^**+**^
	g, *R. salina*	0.0548		**0.0382**^**+**^		− 0.0122		**0.0033**		0.0103		**0.0120**	
	µ, *C. weissflogii*	0.0585	0.00	**0.0582**	**0.17**	0.0417	0.24	**0.0043**	**0.01**	0.0474	0.27	**0.0224**	**0.09**
	g, *C. weissflogii*	0.0014		**0.0152**		0.0131		**0.0025**		0.0092		**0.0068**	
	µ, *M. rubrum*	− 0.0225	0.17	**− 0.0028**	**0.11**	0.0002	0.10	**− 0.0097**	**0.49**	− 0.0073	0.01	**− 0.0073**	**0.00**
	g, *M. rubrum*	− 0.0266		**− 0.0275**		0.0089		**0.0168**		− 0.0030		**0.0020**	
	µ, *S. arenicola*	− 0.0540	0.33	**− 0.0204**	**0.00**	0.0625	0.28	**0.0404**	**0.03**	0.0235	0.08	**0.0200**	**0.07**
	g, *S. arenicola*	− 0.0322		**− 0.0024**		0.0243		**− 0.0098**		0.0053		**− 0.0075**	

The significance of the slope of the regressions is also listed.

Columns in bold correspond to the D treatment whereas the remaining are relative to the L/D ones.

Values marked with an ^#^ showed saturation and g was then calculated according to Gallegos^74^.

R^2^ values marked with a ^+^ or ^++^ are significant, i.e., *P* < 0.05 and *P* < 0.01 respectively.

**Figure 2:**
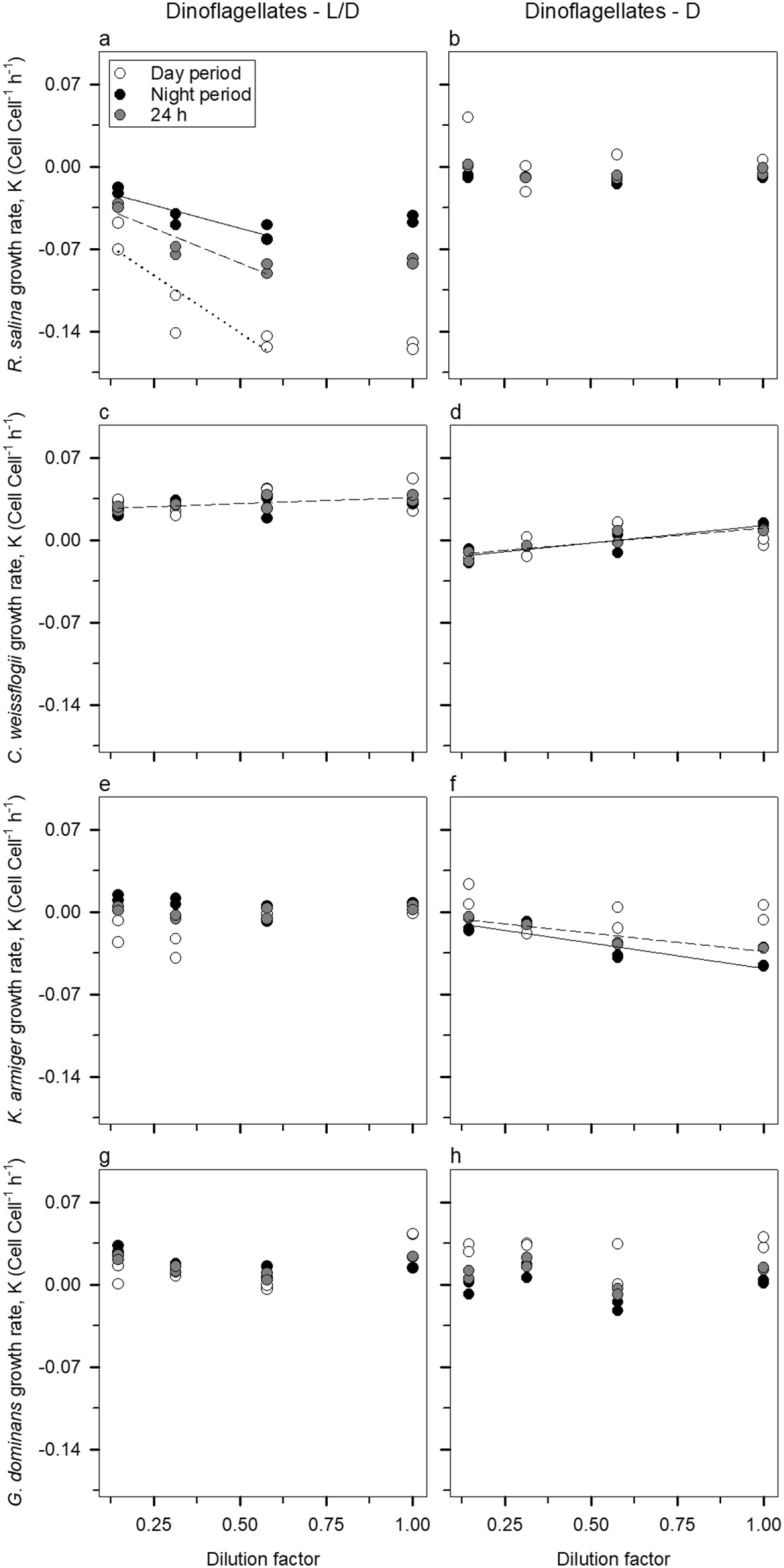
Cell-based dilution grazing experiment with dinoflagellates. The left panels (**a**, **c**, **e**, and **g**) depict the L/D bottles and the right ones (**b**, **d**, **f**, and **h**) are relative to the D bottles. Plotted regressions imply a significant slope (*P* < 0.05). Dotted regression lines, dashed regressions lines, and solid regression lines correspond to the day period, integrated 24 h incubations, and night period, respectively.

**Figure 3:**
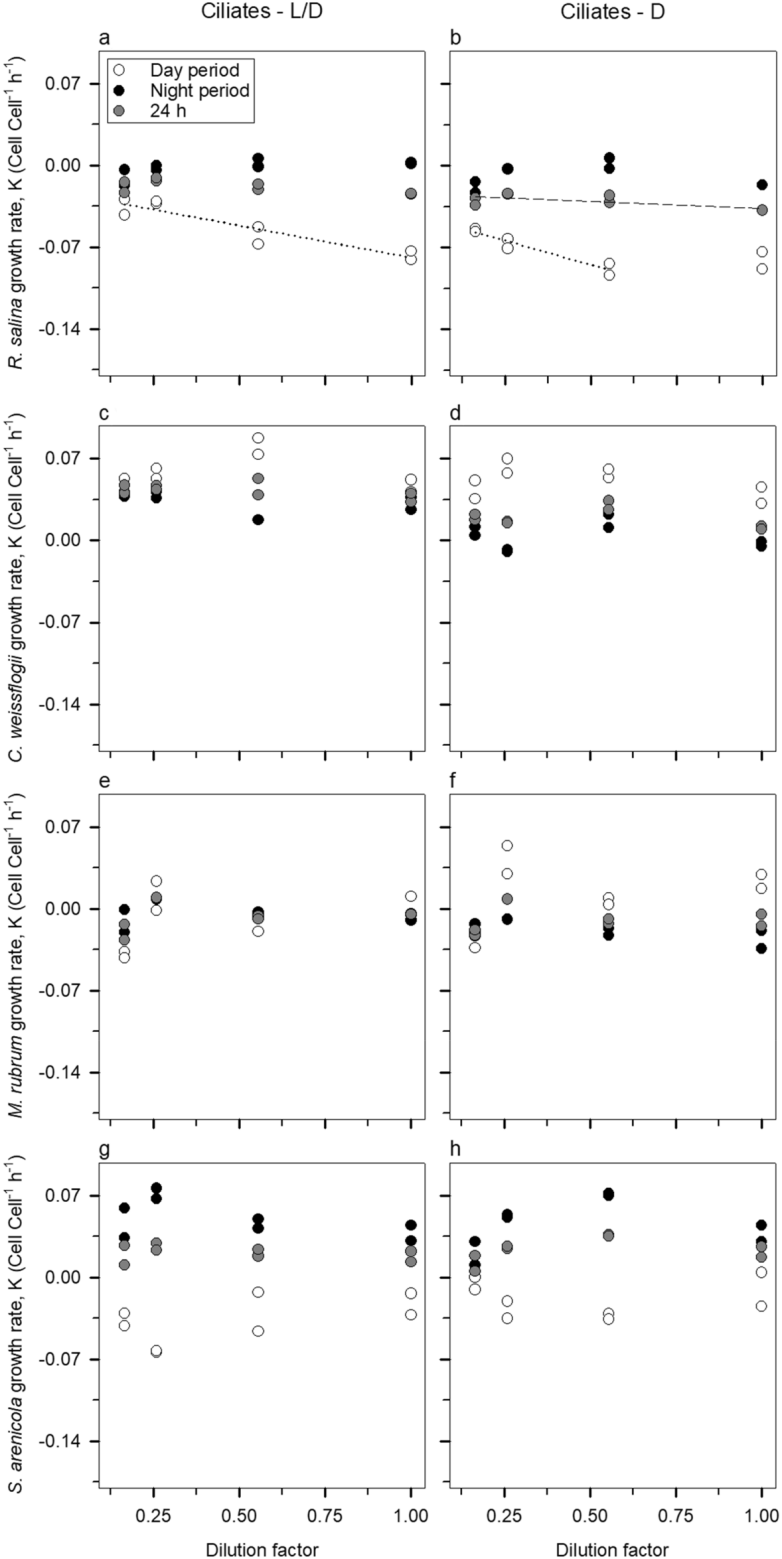
Cell-based dilution grazing experiment with ciliates. Legend as in Fig. 2.

Cell-based dilution regressions for dinoflagellates showed very distinct patterns for the two prey (Fig. 2). *R. salina* (Fig. 2a, b) was always ingested irrespective of the period of the day and light conditions (although it had higher grazing mortality during the day in the presence of light), but the diatom *C. weissflogii* was not (Fig. 2c, d). In fact, the diatom seemed to benefit from the presence of predators, as suggested by the significantly positive slopes both in the regular diel light cycle (hereafter termed L/D) and complete darkness (hereafter termed D) treatments (see the “Methods” section for the experimental conditions of each treatment). When the predator community was composed of ciliates instead of dinoflagellates (Fig. 3), *R. salina* was subject to significant grazing mortalities (i.e., negative slope) during the day in both L/D and D treatments, and in the integrated 24 h in the D treatment (Fig. 3a, b).

All four species of predators showed a lack of response of growth rates to the dilution of the community (Figs. 2e, g, h and 3e–h), except for *K. armiger* in the D bottles (Fig. 2f). It seems then that *K. armiger* was actively ingested by *G. dominans* in the D treatments as ascertained by the significant grazing mortality (*P* < 0.05).

### Incubation experiments

By having control bottles held under the same conditions as the dilution series, we were able to determine individual grazing rates for each predator species. Therefore, it was possible to (1) calculate the individual ingestion rate of each predator on both prey (Table 2), (2) combine the previous information to estimate what would be the merged ingestion rate per pair of predators, (3) calculate the actual ingestion rate for each pair of grazers by comparing data from the 100% bottles and controls without grazers, and (4) calculate the ingestion rates based on the slopes of the dilution regressions.

**Table 2:** Carbon-specific ingestion rates (pg C pg C^−1^ h^−1^) for each predator on both prey items as ascertained by the control bottles with a single predator.

Predator	Prey	Ingestion rates (pg C pg C^−1^ h^−1^)			
		Day	Day	Night	Night
*G. dominans*	*R. salina*	0.17 ± 0.02^a^	**0.02 ± 0.01**^**b**^	0.02 ± 0.00^b^	**NS**
	*C. weissflogii*	− 0.02 ± 0.01^a^	**0.00 ± 0.00**^**a**^	NS	**− 0.01 ± 0.00**^**a**^
*K. armiger*	*R. salina*	0.04 ± 0.00^a^	**0.03 ± 0.00**^**a**^	0.04 ± 0.00^a^	**NS**
	*C. weissflogii*	− 0.02 ± 0.01^a^	**NS**	0.02 ± 0.00^a^	**− 0.01 ± 0.00**^**a**^
*S. arenicola*	*R. salina*	0.16 ± 0.01^a^	**0.21 ± 0.00**^**b**^	0.06 ± 0.00^c^	**0.02 ± 0.00**^**d**^
	*C. weissflogii*	− 0.04 ± 0.00^a^	**0.02 ± 0.01**^**a**^	0.02 ± 0.01^a^	**− 0.04 ± 0.03**^**a**^
*M. rubrum*	*R. salina*	0.03 ± 0.02^a^	**NS**	− 0.02 ± 0.00^b^	**0.01 ± 0.01**^**a,b**^
	*C. weissflogii*	− 0.10 ± 0.01^a^	**− 0.08 ± 0.06**^**a**^	NS	**− 0.07 ± 0.03**^**a**^

NS implies that the measured ingestion rate was not significantly different from 0.

Columns in bold correspond to the D treatment whereas the remaining are relative to L/D.

Different letters within a given prey row imply statistically significant differences between treatments (one-way ANOVA, Tukey HSD, *P* < 0.05).

Most comparisons resulted in non-significant (i.e., not different from 0, two-tailed Student’s t-test, *P* > 0.05) ingestion rates over *C. weissflogii*, except for *S. arenicola* in some treatments, and *K. armiger* during the night-time in the L/D treatment (Table 2). In the L/D treatment, *G. dominans* consumed more *R. salina* during the day than at night (Tukey HSD, *P* < 0.05), a pattern shared by all grazers except *K. armiger*, whose differences between day and night periods were negligible. *M. rubrum* was the species with the largest day/night differences, as it was the only species displaying a significantly negative ingestion rate on *R. salina* during the night.

The D treatments affected the grazers differently: *G. dominans* and *K. armiger* decreased their ingestion rates during the day (despite being significant only in the former) and displayed non-significant ingestion rates at night. Conversely, *S. arenicola* benefitted from the D treatment during the day (Tukey HSD, *P* < 0.05) despite having its ingestion rate decreased during the night (Tukey HSD, *P* < 0.05). Finally, ingestion rates by *M. rubrum* decreased to negligible levels during the daytime in the D treatments (two-tailed Student’s t-test, *P* > 0.05). The night ingestion rates of the D treatment were significantly positive whereas the same period in L/D yielded significantly negative ingestion rates (Table 2) however, this difference was not significant due to the variability of the data (Tukey HSD, *P* > 0.05). Protozooplankton displayed higher 24 h integrated ingestion rates on *R. salina* than did mixoplankton regardless of the light conditions. This difference was more evident in the presence of light but not negligible in its absence. In the L/D treatment, *G. dominans* exhibited carbon-specific ingestion rates ca. 1.5 times higher than *K. armiger*, and *S. arenicola* completely outcompeted *M. rubrum* with an ingestion rate ca. 21.4 times superior. In the D treatment, the differences were lessened to ca. 1.3 and 6.7 times, respectively for dinoflagellates and ciliates.

**Figure 4:**
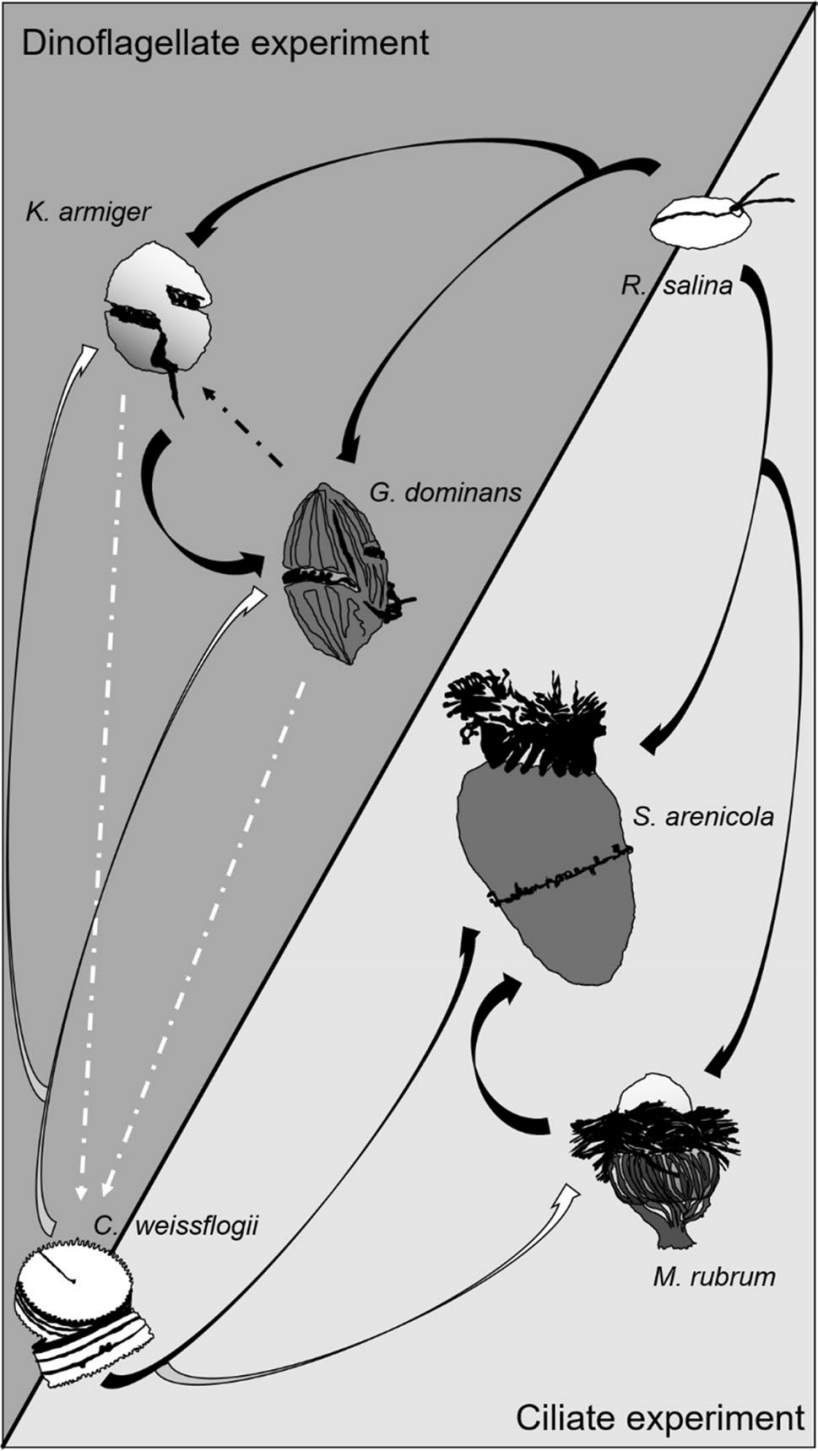
Schematic representation of the interactions found between the protist species used in the present study. Phytoplankton species are depicted in white and protozooplankton in dark grey. Mixoplankton are shown in a gradient tone between the latter two. The tip of the arrow points to the organism benefitting from the interaction. Thick black arrows mean that the ingestion was observed in the dilution grazing experiment, whereas thick white arrows denote that the ingestion was expected (based on previous trials using single predator–prey interactions) but not confirmed. The black dashed arrow implies allelopathy. The white dashed arrows between the dinoflagellates and the diatom indicate that the latter benefits from the presence of these predators though the exact mechanism behind this interaction is unknown.

A diagram that summarises the interactions found between our protist species can be found in Fig. 4. On this conceptual model, we can see the trophic interactions that took place in our experiments. In accordance to our pre-experiment trials, we expected to find ingestion on the cryptophyte *R. salina* and on the diatom *C. weissflogii* by all grazers. Indeed, we were able to quantify ingestion rates on the cryptophyte by all predator species studied, and in all the light conditions tested. However, ingestion of the diatom was only detected for *S. arenicola*. Unexpectedly, the diatom even seemed to benefit from the combined presence of the grazers in the dinoflagellate experiment. Finally, we confirmed that the protozooplanktonic predators within each experiment were able to feed on their mixoplanktonic counterparts although *K. armiger* decreased the growth rates of its competitor, *G. dominans*, likely due to toxicity.

**Figure 5:**
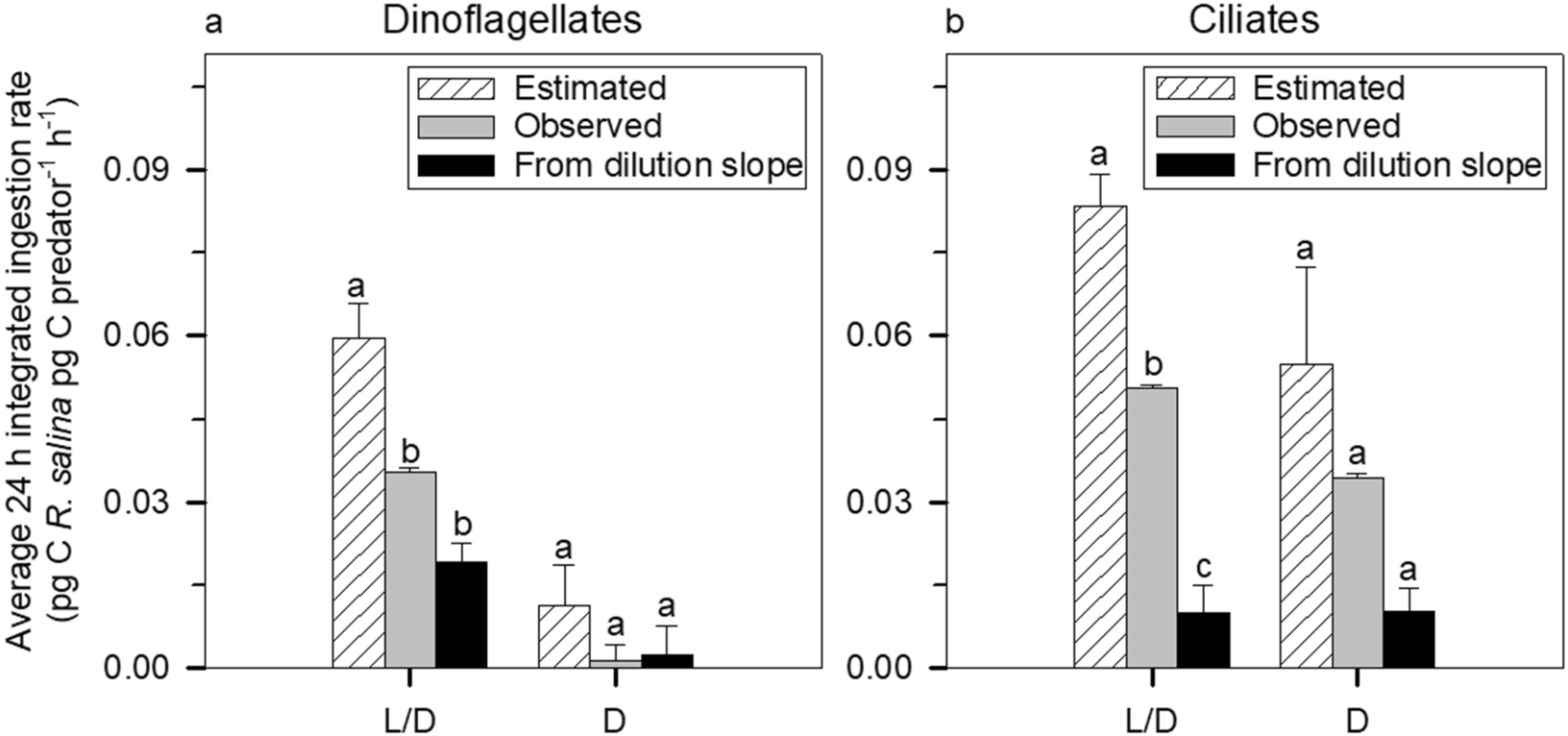
Comparison between estimated (), observed (), and dilution-measured (■) ingestion rates (pg C *R. salina* pg C predator^−1^ h^−1^) in the L/D and D bottles over a 24 h period: (**a**) experiment with dinoflagellates and (**b**) experiment with ciliates. Notice that dilution-measured ingestion rates were calculated using g values listed in Table 1. See the Methods for a detailed explanation of the calculation of each value. Different letters within each group of bars (i.e., L/D evaluated independently from D bottles) imply statistically significant differences (one-way ANOVA, Tukey HSD, *P* < 0.05).

The integrated 24 h period grazing for each predator tandem calculated as explained before is summarised in Fig. 5. Since *C. weissflogii* was often not consumed in the experiments, we have shown only the data regarding *R. salina*. The estimated ingestion rates (obtained from the grazing impact of each individual grazer) were higher than those measured in the undiluted bottles against the respective controls. Additionally, ingestion rates calculated from the dilution slope (without controls) tend to be lower than those measured using the control bottles containing both grazers. However, the differences between methods used to ascertain ingestion rates were only significant in the L/D treatments.

**Figure 6:**
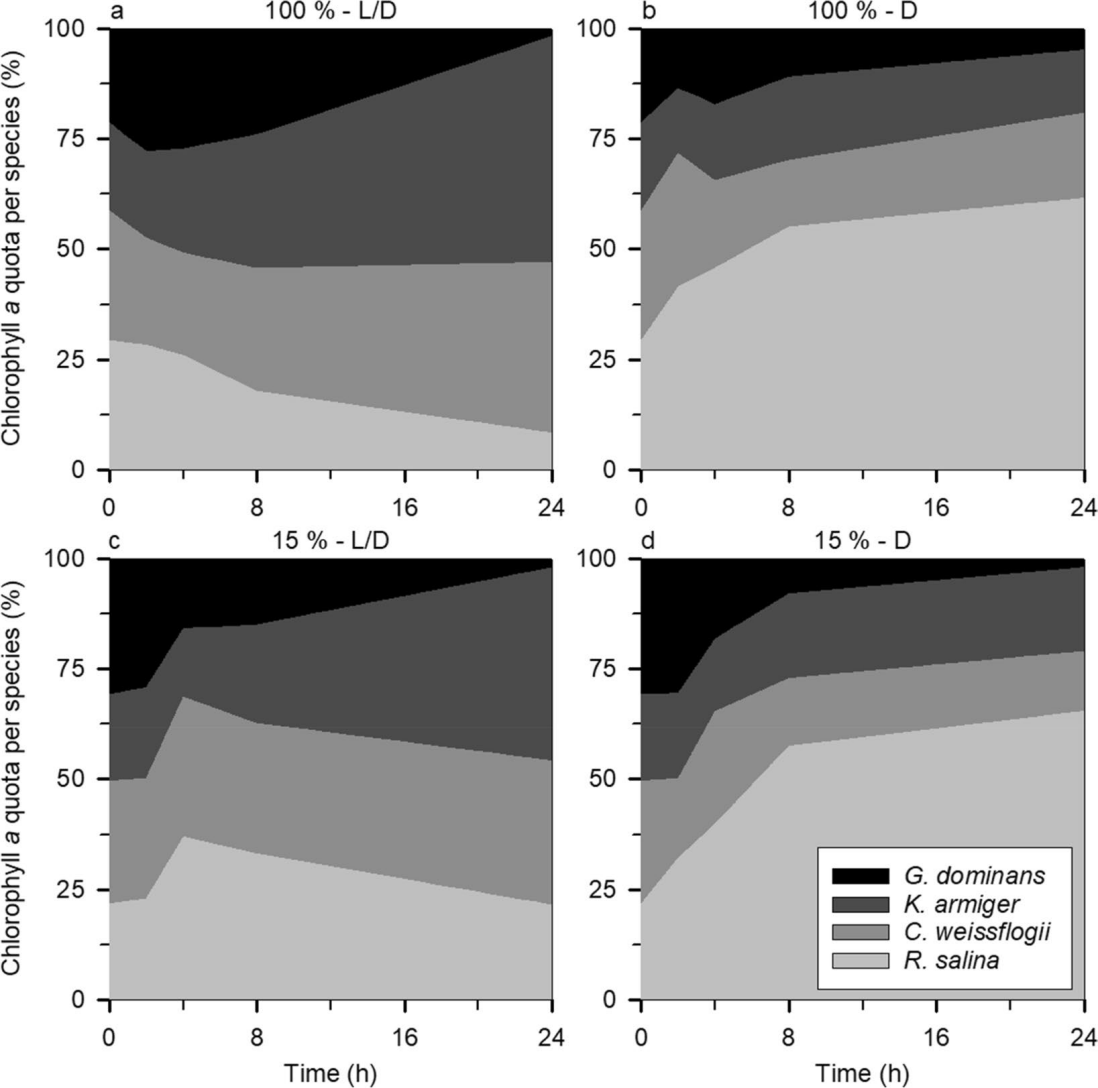
The proportion of the total Chl *a* (%) represented by each species (in different colours) in the dinoflagellate experiment throughout the incubation: (**a** and **c**) L/D treatment with the undiluted and most dilute communities respectively; (**b** and **d**) D treatment with the undiluted and most dilute communities respectively.

**Figure 7:**
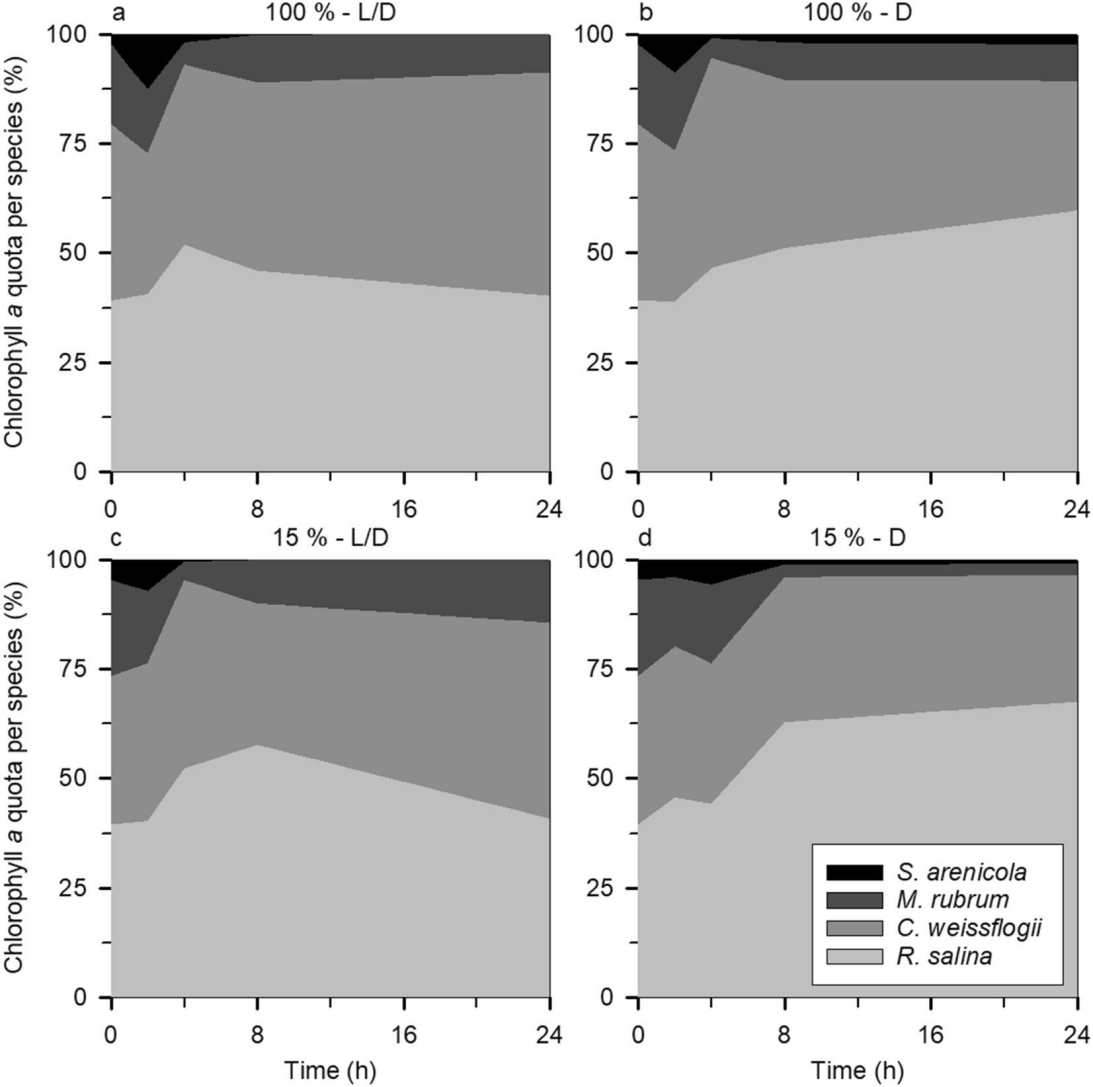
The proportion of the total Chl *a* (%) represented by each species (in different colours) in the ciliate experiment throughout the incubation. Legend as in Fig. 6.

To further understand the Chl *a* dynamics that shaped the outcome of the dilution experiments based on this proxy, we evaluated the contribution of each species to the total Chl *a* pool (Figs. 6 and 7) both in the undiluted and most diluted treatments. Regarding the dinoflagellate experiment (Fig. 6), both the diatom and *K. armiger* became more relevant to the total Chl *a* as time passed, in particular in the undiluted L/D treatment (Fig. 6a) where they increased their contribution to the total Chl *a* by ca. 9.3 and 31.7% respectively. The D treatment has a completely different pattern, with *R. salina* benefiting the most, in particular when the predator concentration was low (Fig. 6d), becoming ca. 65.4% of the total Chl *a* of the system at the end of the incubation (as compared to 21.8% in the beginning). Irrespective of the light conditions, *G. dominans* displayed a particularly significant contribution to the total Chl *a* (up to 30.8%) at the beginning of the incubation. The experiment with ciliates (Fig. 7) followed a similar trend for the diatom and the protozooplankton (Fig. 7a), albeit to a slightly larger extent in the former (an increase of ca. 10.9%) and smaller in the latter (maximum contribution of ca. 12.7%). *M. rubrum*, in contrast with its dinoflagellate counterpart, decreased its contribution to the total Chl *a* by ca. 9.2% (Fig. 7a). Concerning the D treatment, *R. salina* was also the species that fared better with an increase of ca. 28.0% in the diluted treatment (Fig. 7d).

In general, when the incubation contained only one predator species, the calculated individual Chl *a* content was, on average, ca. 8.5% higher than when two predators were incubated together. Additionally, the magnitude of this effect differed between undiluted bottles, and the most diluted treatments (raw data not shown but incorporated in Figs. 6 and 7).

## Discussion

Our results show that Chl *a* alone is not an adequate proxy for prey growth rates in dilution grazing experiments when mixoplankton are present^5,10^. Chlorophyll is, in any case, a poor proxy for phototrophic plankton biomass^31^ because of inter-species variations, and also for the photoacclimation abilities of some species (for which very significant changes can occur within a few hours). The problem extends to the involvement of mixoplanktonic prey and grazers. Nevertheless, even very recent studies continue to rely on this parameter for quantifications of grazing despite acknowledging the dominance, both in biomass and abundance, of mixoplanktonic predators in their system^30^. Moreover, the detailed analysis of the species-specific dynamics revealed that different prey species are consumed at very different rates. In our experiments, and contrary to expectations (see^32,33^, and Fig. S1 in the Supplementary Information), *C. weissflogii* was only actively ingested in the ciliate experiment and, according to the results from the control bottles (Table 2), not by *M. rubrum* (see Fig. 4 and Fig. S1a).

Certainly, it is not the first time that a negative selection against diatoms has been seen; for example, Burkill et al.^34^ noticed that diatoms were less grazed by protist grazers than other phytoplankton species, as assessed by a dilution technique paired with High-Performance Liquid Chromatography for pigment analysis. Using the same method, Suzuki et al.^35^ reported that diatoms became the dominant phytoplankton group, which suggests that other groups were preferentially fed upon. Calbet et al.^36^, in the Arctic, also found only occasional grazing over the local diatoms. In our study, diatoms were not only not consumed, but the presence of dinoflagellates appeared to contribute to their growth (Fig. 4), this relationship being partly dependent on the concentration of the predator (see Fig. 2c, d). This result could be a direct consequence of assimilation and use of compounds (e.g.,^37,38^) released by microplankton such as ammonium (e.g.,^39,40^) and urea (e.g.,^41^), which were not supplied in the growth medium, but which would have supported prey growth. Alternatively, this unexpected outcome may have been a consequence of the selective ingestion of *R. salina* by the two predators, relieving the competition for nutrients and light and resulting in a higher growth rate of the diatom in the presence of the predators. We cannot rule out the fact that diatoms sink faster than flagellates which, as the bottles were not mixed during most of the incubation period (although gently mixed at every sampling point), may have also involuntarily decreased ingestion rates on *C. weissflogii*. Still, one *C. weissflogii* cell contains, on average, ca. 2.5 times more Chl *a* than one *R. salina* cell (initial value excluded, see Table 3). Taken together with the preference for *R. salina* it is not surprising that the proportion of total Chl *a* represented by the diatoms increased over time, in particular in the L/D treatment (Figs. 6a, c and 7a, c).

**Table 3:** Chl *a* content (pg Chl *a* cell^−1^) of the target species at each sampling point as calculated from the control bottles.

Species	Sampling points				
	t = 0 h	t = 8 h	t = 8 h	t = 24 h	t = 24 h
*R. salina*	0.63	1.66 ± 0.11	**1.38 ± 0.17**	1.22 ± 0.23	**1.58 ± 0.11**
*C. weissflogii*	5.59	4.65 ± 0.66	**3.40 ± 0.35**	3.07 ± 0.51	**3.30 ± 1.37**
*K. armiger*	6.71	10.94 ± 0.05	**6.70 ± 0.27**	17.38 ± 0.80	**8.16 ± 0.13**
*M. rubrum*	19.98	21.97 ± 1.14	**12.50 ± 2.82**	19.97 ± 2.09	**15.22 ± 0.74**
*G. dominans*	19.88	15.23 ± 3.90	**6.69 ± 0.52**	2.74 ± 0.78	**1.84 ± 4.58**
*S. arenicola*	10.22	0.62 ± 6.44	**13.06 ± 0.24**	0.00^+^	**4.52 ± 1.13**

Columns in bold correspond to the D treatment whereas the remaining relate to the L/D one.

The initial samples were the same for both treatments.

The ^+^ implies that the calculations yielded a negative value and, as this is an impossible solution, forced the value to be 0.

Another factor clearly highlighted by our experiments, is that protozooplankton themselves contribute a significant portion of the total chlorophyll of the system (due to ingested Chl *a*), in particular at the beginning of the incubation (see Figs. 6 and 7); this being invariably ignored in a traditional dilution experiment. The high Chl *a* detected inside the protozooplanktonic grazers at the beginning of the incubations could suggest that the system was initially not in equilibrium, and that this was the result of superfluous feeding (e.g.,^42^). This would, nevertheless, be surprising since we required ca. 1 h to collect the initial samples (t = 0 h) after joining all the organisms together (see the section “Dilution grazing experiments” in the “Methods” section); previous studies, like the one on *G. dominans* and *Oxyrrhis marina* by Calbet et al.^42^, showed that the hunger response and consequent vacuole replenishment occurred in ca. 100 min for very high prey concentrations and it is expected to decrease at lower prey concentrations as the ones used in our study. Therefore, even if one assumes that the first 4 h of incubation are a result of superfluous feeding, after 24 h, the “estimated”, “observed”, and “from dilution slope” grazing estimates are not significantly different to those displayed in Fig. 5 (*P* > 0.05 in all instances) and, therefore, we can assume that the hunger response was likely irrelevant (e.g.,^43^) and did not mask our results. In any case, as stated before, an actual field grazing dilution experiment also suffers from similar problems, because grazers and prey are suddenly diluted and not pre-adapted to distinct food concentrations. Nevertheless, this is not novel information, since Chl *a* and its degradation products have been found inside several protozooplankton species from different phylogenetic groups immediately after feeding^44^ and even after some days without food^45^. An increase in intracellular Chl *a* concentrations immediately after feeding has also been found in mixoplankton^46,47^, on which this increase is derived both from ingested prey as well as from new synthesis of their own Chl *a*. Additionally, several experiments with Live Fluorescently Labelled Algae (LFLA) show that predators (irrespective of their trophic mode) seem to maximise the concentration of intracellular prey shortly after the initiation of the incubation (e.g.,^48^; Ferreira et al., submitted). Indeed, some authors have even been able to measure photosynthesis in protozooplankton, like the ciliates *Mesodinium pulex*^49^ and *Strombidinopsis* sp.^50^.

The fact that Chl *a* is a poor indicator of phytoplankton biomass and the inherent consequences discussed so far can be solved by the quantification of the prey community abundance (e.g.,^51^) by microscopy or by the use of signature pigments for each major phytoplankton group. The latter method, however, is not as thorough as the former, since rare are the cases where one pigment is exclusively associated with a single group of organisms (see^52^ and references therein). In any case, any pigment-based proxy is subject to the same problems, as identified by Kruskopf & Flynn^31^. Irrespective of the quantification method, it has been made evident that the different algae are consumed at different rates (e.g., pigments^10,34,35^; microscopy^5,36^).

Prey selection in protistan grazers is a common feature (e.g.,^23,26–28^). Given the diversity of grazers in natural communities and the array of preferred prey that each particular species possesses, it is logical to think that dilution experiments will capture the net community response properly. Likewise, grazers interact with each other through toxins, competition, and intraguild predation among other factors. An example of intraguild predation could be the observed on *K. armiger* by *G. dominans* (see Figs. 2f and 4 and Table 1), which caused an average loss of ca. 18.72 pg of *K. armiger* carbon per *G. dominans* per hour in the D treatment. Interestingly, in the same treatment, a slight negative effect of *K. armiger* on its predator *G. dominans* can also be deduced (i.e., positive g, Table 1), resulting in an average loss of ca. 0.33 pg *G. dominans* carbon per *K. armiger* per hour. This could be a consequence of algal toxins, since *K. armiger* is a known producer of karmitoxin^22^, whose presence may have negative effects even on metazoan grazers^21^. Regarding ciliates, none of the species used is a known producer of toxic compounds, which suggests that the average loss of ca. 1.25 pg M*. rubrum* carbon per hour in the D treatment was due to *S. arenicola* predation. Altogether, it seems clear from our data that intraguild predation cannot be ignored when analysing dilution experiments (Fig. 4). Furthermore, our results clearly show that single functional responses cannot be used to extrapolate community grazing impacts, as evidenced by the differences in estimated and measured ingestion rates based on the disappearance of prey in combined grazers experiments (Fig. 5). Nevertheless, this is a relatively common procedure (e.g.,^53^ and references therein). Often in modelling approaches, individual predator’s functional responses have been used to extrapolate prey selectivity and community grazing responses^27^; in reality complex prey selectivity functions are required to satisfactorily describe prey selectivity and inter-prey allelopathic interactions^54^.

It is, however, also evident that the measured ingestion rates in combined grazers experiments were not the same as those calculated from the slope of the dilution grazing experiment. This raises the question of why was that the case. It is well known that phytoplankton cultures, when extremely diluted, show a lag phase of different duration^55^ which has been attributed to the net leakage of metabolites^56^. Assuming that the duration of the lag phase will be dependent on the level of dilution, it seems reasonable to deduce that after ca. 24 h the instantaneous growth rates (µ) in the most diluted treatments will be lower than that of the undiluted treatments. This has consequences, not only for the estimated prey growth rates but also for the whole assessment of the grazing rate, due to the flattening of the regression line (i.e., the decrease in the computed growth rate). This artefact may be more evident in cultures acclimated to very particular conditions (as the laboratory cultures used in this study) than in nature.

Another important finding of our research is the importance of light on the correct expression of the feeding activity by both mixoplankton and protozooplankton. We noticed that irrespective of the light conditions, all species exhibited a diurnal feeding rhythm (*R. salina* panels in Figs. 2 and 3), which is in accordance with earlier observations on protists (e.g.,^29,57,58^). The presence of light typically increased the ingestion rates. Additionally, the ingestion rates differed during the night period between L/D and D treatments, which implies that receiving light during the day is also vital in modulating the night behaviour of protoozoo- and mixoplankton. In particular, mixoplankton grazing is usually affected by light conditions, typically increasing (e.g.,^32,59^), but also sometimes decreasing(e.g.,^60^) in the presence of light. Different irradiance levels can also affect the magnitude of ingestion rates both in protozoo- and mixoplankton (see^61^ and references therein).

For those reasons, we hoped for a rather consistent pattern among our protists that would help us discriminate mixoplankton in dilution grazing experiments. As a matter of fact, based on the results from Arias et al.^29^, we expected that in the dinoflagellate experiment, the D treatment would have inhibited only the grazing of *K. armiger*, enabling a simple discrimination between trophic modes. The reality did not meet the expectations since the day and night-time carbon-specific ingestion rates (as assessed using the control bottles, Table 2) of *K. armiger* were respectively higher and equal than those of *G. dominans*. Conversely, in the ciliate experiment, protozooplankton were the major grazers in our incubations regardless of the day period and light conditions. This response was not as straightforward as one would expect it to be because *M. rubrum* has been recently suggested to be a species complex containing at least 7 different species (^62^ and references therein), which hinders any possible conjecture on their grazing impact. Indeed, the uneven responses found between and within trophic modes precluded such optimistic hypothetical procedure.

The D treatment in the present paper illustrated the importance of mimicking natural light conditions, a factor also addressed in the original description of the technique by Landry and Hassett^1^. It is crucial for the whole interpretation of the dilution technique that incubations should be conducted in similar light (and temperature) conditions as the natural ones to allow for the continued growth of the phototrophic prey. However, here we want to stress another aspect of the incubations: should they start during the day or the night? Considering our (and previous) results on diel feeding rhythms, and on the contribution of each species to the total Chl *a* pool, it is clear that different results will be obtained if the incubations are started during the day or the night. Besides, whether day or night, organisms are also likely to be in a very different physiological state (either growing or decreasing). Therefore, we recommend that dilution experiments conducted in the field should always be started at the same period of the day to enable comparisons (see also Anderson et al.^14^ for similar conclusions on bacterivory exerted by small flagellates). Ideally, incubations would be started at different times of the day to capture the intricacies of the community dynamics on a diel cycle. Nevertheless, should the segmented analysis be impossible, we argue that the right time to begin the incubations would be during the night, as this is the time where ingestion rates by protozooplankton are typically lower (e.g.,^29,57,58^, this study) and would, consequently, reduce their quota of Chl *a* in the system.

Lastly, we want to stress that we are aware that our study does not represent natural biodiversity because our experiments were conducted in the laboratory with a few species. Nevertheless, we attempted to use common species of wide distribution for each major group of protists to provide a better institutionalisation of our conclusions. Further to the choice of predator and prey is their concentrations and proportions. Being a laboratory experiment designed to understand fundamental mechanisms within a dilution grazing experiment, we departed from near saturating food conditions from where we started the dilution series. In nature, the concentrations that we used may be high but are not unrealistic, and actually lower than in many bloom scenarios. We included diatoms at high concentrations, even knowing that they are not the preferred prey of most grazers^34^, because diatoms are very abundant in many natural ecosystems and to stress the point of food selection within the experiment. For sure, using different proportions of prey would have rendered different results. However, as previously mentioned, our aim was not to seek flaws in the dilution technique, but to understand the role of mixoplankton in these experiments and the complex trophic interactions that may occur within. Ultimately, with our choice of prey and their concentrations, we have proven that when there is no selection for a massively abundant prey, the use of Chl *a* as a proxy for community abundances may underestimate actual grazing rates.

Some other aspects of our experiments may also be criticised because they do not fully match a standard dilution experiment. For instance, we manipulated light, adding complexity to the study. However, this manipulation enabled the deepening into the drivers of the mixoplanktonic and protozooplanktonic grazing responses. Another characteristic, perhaps awkward, of our study is that we allowed the grazers to deplete their prey before starting the experiment. One may argue this procedure does not mimic the natural previous trophic history a grazer may have in nature. Yet, in nature, when facing a dilution experiment, it is impossible to ascertain whether the organisms are encountering novel prey or not. Indeed, they (prey and predator) could have just migrated into such conditions, or be subject to famine, or just moved from a food patch. In any case, it is true that a consistent “hunger response” would have affected our initial grazing values, biasing grazing rate estimates. To overcome this artefact, we let the grazers feed for about one hour before starting the actual dilution assay (see the “Methods” section). From that point on, any dilution is, in fact, an abrupt alteration of the food scenario, which is likely more important than the previous trophic history of the grazer.

In summary, with these laboratory experiments, we have presented evidence calling for a revision of the use of chlorophyll in dilution grazing experiments^5,10^, and we have highlighted the need to observe the organismal composition of both initial and final communities to better understand the dynamics during the dilution grazing experiments^51^. This approach will not incorporate mixoplanktonic activity into the dilution technique per se however if combined with LFLA (see^5,17^), a semi-quantitative approach to disentangle the contribution of mixoplankton to community grazing could be achieved (although not perfect). An alternative (and perhaps more elegant) solution could be the integration of the experimental technique with in silico modelling. The modelling approaches of the dilution technique have already been used, for example, to disentangle niche competition^63^ and to explore nonlinear grazer responses^20^. We believe that our experimental design and knowledge of the previously indicated data could be of use for the configuration of a dilution grazing model, which could then be validated in the field (and, optimistically, coupled to the ubiquitous application of the dilution technique across the globe). We cannot guarantee that having a properly constructed model that mimics the dilution technique will be the solution to the mixoplankton paradigm. However, it may provide a step towards that goal as it could finally shed much-needed light on the mixo- and heterotrophic contributions to the grazing pressure of a given system. To quote from the commentary of Flynn et al.^6^, it could provide the answer to the question of whether mixoplankton are de facto “another of the Emperor’s New Suit of Clothes” or, “on the other hand (…) collectively worthy of more detailed inclusion in models”.

## Methods

We constructed several artificial food chains involving protozooplanktonic and mixoplanktonic predators to gain insights into the dynamics of dilution grazing experiments. Being a laboratory experiment, we were able to control variables and unknowns that cannot be controlled in field experiments. In particular, we included prey controls and ascertained single grazer rates at the experimental conditions. These additions enabled us to determine the species-specific contributions to the concentration of chlorophyll and grazing in the mixed dilution grazing experiment. We conducted our experiments with and without light and sampled the bottles at several time points to have a better representation of the predator–prey dynamics during the incubations.

### Cultures

We conducted the experiments with the protozooplanktonic dinoflagellate *Gyrodinium dominans* (strain ICM-ZOO-GD001), the protozooplanktonic ciliate *Strombidium arenicola* (strain ICM-ZOO-SA001), the Constitutive mixoplankton^6,7^ dinoflagellate *Karlodinium armiger* (strain ICM-ZOO-KA001), and the Non-Constitutive mixoplanktonic^6,7^ ciliate *Mesodinium rubrum* (strain DK-2009). As prey for all experiments, we used the cryptophyte *Rhodomonas salina* (strain K-0294) and the diatom *Conticribra weissflogii* (previously known as *Thalassiosira weissflogii*, strain CCAP 1085/18). *R. salina* was chosen as prey since it is known to be actively ingested by all the chosen predators (e.g.,^64^). Furthermore, *K. armiger* is known to feed on *C. weissflogii*^32^ and *G. dominans* can ingest and grow on *Conticribra* sp.^33^. Before conducting the dilution grazing experiments, a trial took place where we incubated all predators with the diatom and examined the samples using epifluorescence microscopy. We found red chloroplasts inside several *M. rubrum* cells after incubating these predators with the diatom (Fig. S1a; under blue light excitation, chloroplasts of *Teleaulax amphioxeia* glow orange due to the presence of phycoerythrin- e.g.,^16^, i.e., red chloroplasts likely belonged to the diatom). In these trials, we also confirmed that *G. dominans* was able to engulf *C. weissflogii* (Fig. S1b) and found no direct evidence of ingestion, neither in *K. armiger* nor in *S. arenicola*.

During the up-scale and pre-experimental periods, all predators were offered *R. salina* as prey except *M. rubrum*, which was given *T. amphioxeia* (strain K-1837) instead. One day before the experiment, we allowed all predators to deplete their prey to extinction.

Both *R. salina* and *T. amphioxeia* were kept in f/2 medium^65^ and irradiated at a photon flux density (PFD) of ca. 150 µmol photons m^−2^ s^−1^ provided by cool white fluorescent lights. *C. weissflogii* was kept under the same conditions although silicate was added to the medium. All predators were kept in autoclaved 0.1 µm-filtered seawater. Protozooplankton were maintained at a PFD of ca. 35 µmol photons m^−2^ s^−1^ whereas mixoplankton were kept at ca. 65 µmol photons m^−2^ s^−1^.

The stock cultures were maintained using a semi-continuous approach, i.e., the cultures were diluted every 1–2 days with the respective fresh medium (between 20 and 50% of the total volume), to maintain them under exponential growth (and within target concentrations) at any moment. Additionally, to avoid an increase in the pH beyond the limits for exponential growth (i.e., without inorganic carbon limitation)^66^, all cultures were bubbled with 0.2 µm-filtered air. We used a very slow cadence of bubbles (flow rate not measured) to diminish the chances of stressing the predators^66^. The direct effect of the bubbling process on the growth of the protists was not determined however, it was likely minor as we confirmed that all cultures were healthy and actively feeding before starting the experiments. All cultures were kept in a controlled-temperature room at 19 °C with a 10:14 L/D cycle at a salinity of 38.

### Dilution grazing experiments

The dilution grazing experiments were conducted with a mixture of predators paired at a time; *G. dominans* paired with *K. armiger*, and *S. arenicola* paired with *M. rubrum* (for a summary of the experimental design, see Table S1 in the Supplementary Information). Both experiments were conducted with a mixture of *R. salina* and *C. weissflogii* as prey, at a similar carbon concentration. The initial concentration of prey on the undiluted bottles was ca. 2.5 × 10^4^ (both species combined) as we were aiming at saturating food conditions for mixoplanktonic predators (see Fig. S2), from where the dilution series began. Carbon concentrations for all species were obtained from the average volume and Carbon: µm^3^ ratio provided by Traboni et al.^67^. All predators were allowed to deplete their co-occurring prey before starting the experiment, to reset their feeding history. In this way, we also ensured a non-acclimated scenario to food conditions, which is typical in standard field dilution experiments, as organisms are never adapted to the dilution itself.

Two dilution series of 60, 30, and 15% were prepared from the 100% treatment, in duplicates, within 1100 mL transparent polycarbonate bottles (Thermo Scientific Nalgene). All bottles contained 200 mL of f/2 medium + Si per litre of suspension to reach a final concentration equivalent to f/10 medium + Si^65^. The actual level of dilution was determined from the initial concentration of prey in each dilution relative to the initial concentration of prey in the 100% treatment. One of the dilution series was incubated with a 10:14 L/D cycle at a PFD of 100 µmol photons m^−2^ s^−1^ (L/D treatment). The second series was wrapped in aluminium foil and covered with an opaque box (i.e., incubated in complete darkness) during the whole period (D treatment). It took ca. 1 h between the preparation of the experimental suspension of organisms and the collection of the initial sample. As such, we avoided the typical hunger response and consequent vacuole replenishment of starved predators^42^, and diminished the consequences of photoacclimation in the chlorophyll content of the phototrophs, as this is an almost immediate process^68,69^. The sampling occasions were the only sources of culture vessel mixing during the incubation.

Additionally, a second, third, and fourth set of duplicated 100% bottles were prepared under the same prey, nutrient, and light conditions mentioned before for the dilution grazing experiments. The second set contained the two prey and no predators (termed 100prey). These bottles were used as control and accounted for the net growth rate (both in cell numbers and Chl *a*) of each prey in the absence of grazing. The third and the fourth set of 100% bottles comprised the two prey and only one of the predators (i.e., in the dinoflagellate experiment, 100gyro or 100karlo; in the ciliate experiment, 100strom and 100meso). These bottles eased the interpretation of the more complex mixed experiment by providing outcomes when only a single predator was present.

All treatments were prepared with a final volume of 1 L per bottle. In the dilution series, the bottles from every dilution level were sampled after 0, 2, 4, 8, and 24 h for both Chl *a* (150 mL) and cell counts (70 mL, 2% acidic Lugol’s solution final concentration). The control bottles were sampled after 0, 8, and 24 h (150 mL for Chl *a* and 50 mL for cell counts). Samples collected after 2 and 4 h were only used to calculate Chl *a* per cell concentrations and are, therefore, not going to be further discussed. For the detailed cell counts and Chl *a* concentrations for each time point, see Figs. S3–S12 in the Supplementary Information. The 8 h samples of both L/D and D bottles were collected immediately before the beginning of the night period (i.e., Day period = 0 to 8 h samples; Night period = 8 to 24 h samples). For the D treatment, this did not imply any change in the light conditions despite effectively representing a day sample.

The samples preserved with acidic Lugol’s solution were stored in the dark at 4 °C for 1–6 months before being counted. After stabilising the samples to room temperature (21 ± 3 °C), the bottles were rotated softly and used to fill 10 mL methacrylate sedimentation chambers. The Utermöhl^70^ method was employed to analyse the samples after 24 h on an inverted microscope (XSB-1A) using a 25 × objective. Each replicate was counted twice, with a minimum of 200 organisms from each species per count.

### Chlorophyll, growth, and grazing analysis

The total chlorophyll *a* (Chl *a*, µg L^−1^) was determined by filtering 150 mL of culture from every bottle as specified above. The samples were collected into dark bottles and filtered through Whatman GF/C glass fibre filters under dim light conditions immediately after collection. The filters were folded in half twice, wrapped in aluminium foil and then kept at − 20 °C for ca. 5 months until the extraction of total chlorophyll with 6 mL of acetone 90%. The extraction was conducted in the dark at 4 °C and lasted ca. 24 h, thus avoiding the need to grind the filters^71^. The samples were measured before and after the addition of 100 µL of HCl 10% (final concentration in the extract ca. 0.05 M) on a Turner Designs Fluorometer^72^ to account for the concentration of phaeophytin. The fluorometer was calibrated with a pure Chl *a* standard (2.13 mg Chl *a* L^−1^) of cyanobacterial origin (DHI, Hørsholm, Denmark).. Phaeopigments (µg L^−1^) were determined by dividing the chlorophyll concentration by the acid factor ratio between fluorescence values before and after acidification.

We determined individual species contribution to the total Chl *a* mathematically for each time point in the control bottles (Table 3). First, 100prey bottles were used to determine Chl *a* contents for *R. salina* and *C. weissflogii*. These concentrations of pigment were then integrated into the controls with one predator and in the dilution series bottles to determine the pigment concentrations within each predator cell. We estimated the intermediate time points (those not directly assessed from control bottles) using linear progression.

Growth, clearance, and grazing rates were calculated for every time point using Frost^19^ equations as modified by Heinbokel^73^. If one uses these equations considering the 100prey bottles as controls and the 100gyro, 100karlo, 100strom or 100meso bottles as experimental, ingestion rates for each individual predator can be obtained. Thus, we expect that, when combined together, the total ingestion rate would be the average of the one calculated for each individual predator. This average yields an estimated value. Alternatively, if the control bottles are the same but one considers the 100% bottles as the experimental (i.e., with both predators together), a calculation of the joint ingestion rate per predator (all predators together) can be determined. Therefore, we considered this value to be the observed ingestion rate in our experiments. Finally, a third estimate of grazing can be obtained by measuring the slope of the linear regression that correlates the fraction of undiluted water and the apparent growth rates based on the changes in the concentration of prey during the incubation^1^. This slope yields the grazing coefficient (g), which can be converted into clearance rates by dividing it by the average predator concentration throughout the incubation. Ingestion rates are obtained by multiplying the average prey concentration by the clearance rate of the predators. This was defined as the dilution-measured ingestion rate.

The results of some incubations denoted the presence of saturated feeding responses. Accordingly, under these circumstances, prey growth rates (µ, Chl *a* Chl *a*^−1^ h^−1^) were determined from the interception of linear regression with the 3 most diluted treatments. The grazing coefficients (g) were then calculated as

$${\text{g}} =\upmu - {\text{K}}$$

where K (Chl *a* Chl *a*^−1^ h^−1^) is the apparent growth rates obtained in the undiluted bottles^74^. We followed the same procedure to determine cell-specific grazing rates with the difference that cell counts were used instead of Chl *a*. For the sake of clarity, in Figs. 1, 2 and 3 we decided to show only the regressions whose slope was significantly different (*P* < 0.05) from zero. Nevertheless, we calculated µ and g for all experiments as recommended by Latasa^75^. These values are summarised in Table 1.

## Supplementary Information

Supplementary Information.
