# Supplementary material for: Mixoplankton interferences in dilution grazing experiments
Source: Sci Rep. 2021 Dec 13;11:23849. doi: 10.1038/s41598-021-03176-0 (PMC8668877; doi:10.1038/s41598-021-03176-0)
Supplement: Supplementary file 1 — Supplementary Information. [file 41598_2021_3176_MOESM1_ESM.pdf]

Mixoplankton interferences in dilution grazing experiments

Guilherme D. Ferreira[1,3*], Filomena Romano[2], Nikola Medić[3], Paraskevi Pitta[2], Per Juel Hansen[3], Kevin J. Flynn[4], Aditee Mitra[5], Albert Calbet[1]

[1]Institut de Ciències del Mar, CSIC, Pg. Marítim de la Barceloneta, 37-49, 08003 Barcelona, Spain

[2]Institute of Oceanography, Hellenic Centre for Marine Research, PO Box 2214, 71003 Heraklion, Greece

[3]Marine Biological Section, University of Copenhagen, DK-3000, Helsingør, Denmark

[4] Plymouth Marine Laboratory, Prospect Place, Plymouth, PL1 3DH, UK

[5]School of Earth and Environmental Sciences, Cardiff University, Park Place, Cardiff CF10 3AT, UK

[*]gduarte@icm.csic.es

Table S1. The experimental design used in the dinoflagellate experiment. The whole community comprised *Gyrodinium dominans* and *Karlodinium armiger* as predators, and *Conticribra weissflogii* and *Rhodomonas salina* as prey. The sampling points include one sample for Chlorophyll *a* and one for cell counts. The ciliate experiment followed the same scheme with the difference that the whole community contained *Strombidium arenicola* and *Mesodinium rubrum* as predators, being the controls adjusted accordingly.

Treatment[1]		Sampling points (hours)				
		0	2	4	8	24
Dilution series[2]	100 %	√	√	√	√	√
	60 %	√	√	√	√	√
	30 %	√	√	√	√	√
	15 %	√	√	√	√	√
Controls	100prey	√			√	√
	100gyro	√			√	√
	100karlo	√			√	√

[1]The same design was used under a regular diel light cycle and complete darkness

[2]Executed with the whole community

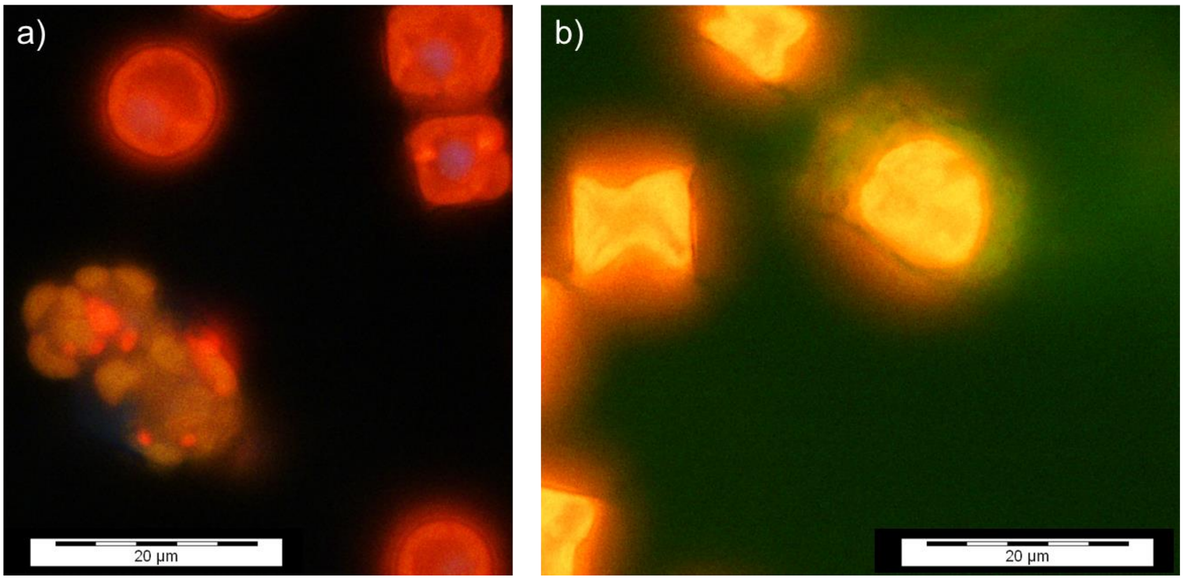

Figure S1. Epifluorescence photographs of a trial conducted before the dilution grazing experiment: a) *Mesodinium rubrum* with red chloroplasts inside (blue light excitation) and b) *Gyrodinium dominans* with a *Conticribra weissflogii* inside (green light excitation). Both images were obtained after incubating both species together for ca. 24 h under regular diel light conditions.

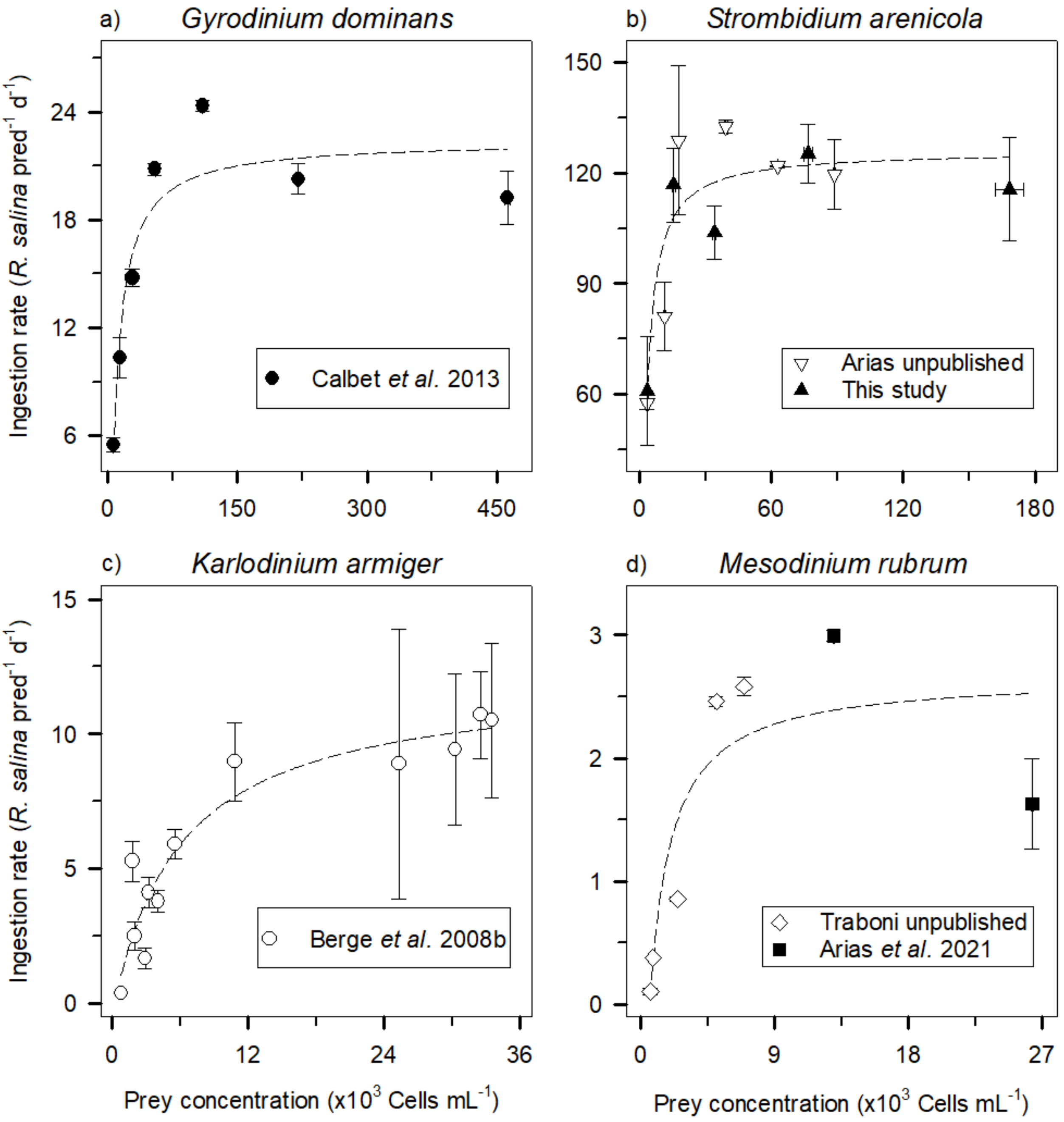

Figure S2 Ingestion rate (Cells predator^{-1} d^{-1}) of a) *Gyrodinium dominans*, b) *Strombidium arenicola*, c) *Karlodinium armiger*, and d) *Mesodinium rubrum* on the cryptophyte *Rhodomonas salina*. The original data source is indicated in each individual panel of the figure. The curve fits were obtained by applying Michaelis-Menten kinetics to the data. Error bars ± se.

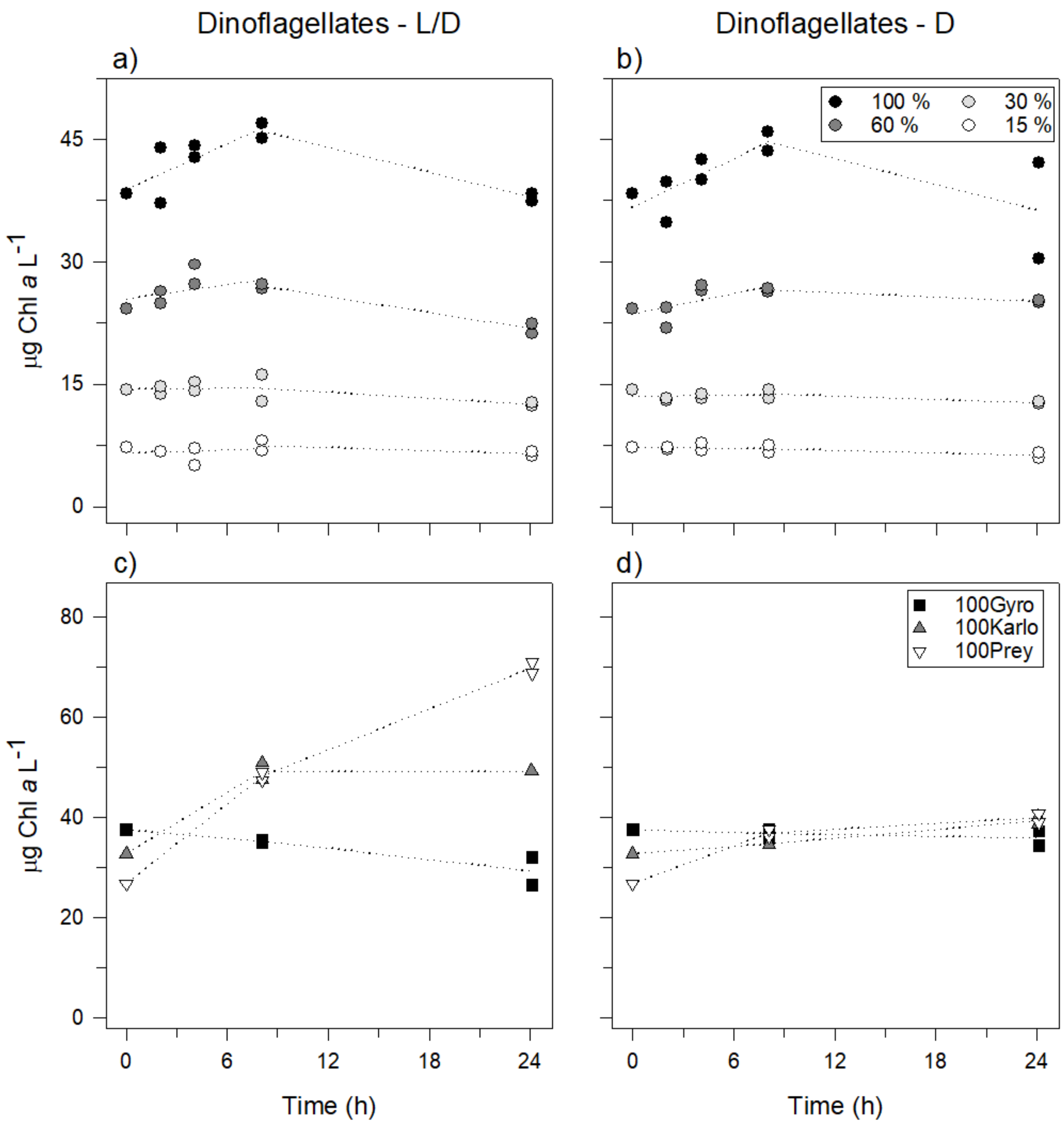

Figure S3. Chl *a* over time for the experiment with dinoflagellates: a and b) dilution series in the L/D and D treatments respectively; c and d) control bottles series in the L/D and D treatments respectively.

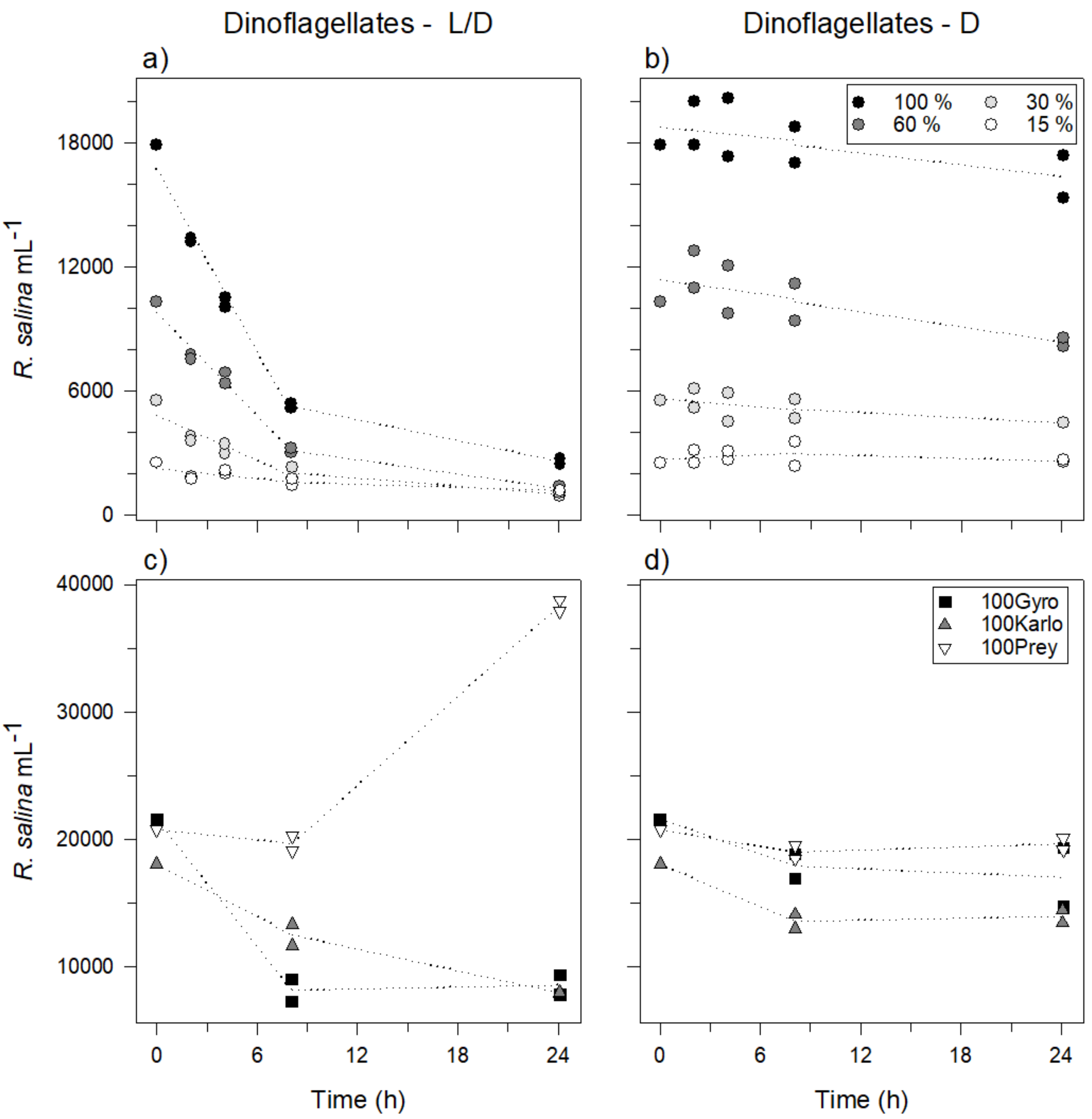

Figure S4. Abundance of *Rhodomonas salina* over time for the experiment with dinoflagellates: a and b) dilution series in the L/D and D treatments respectively; c and d) control bottles series in the L/D and D treatments respectively.

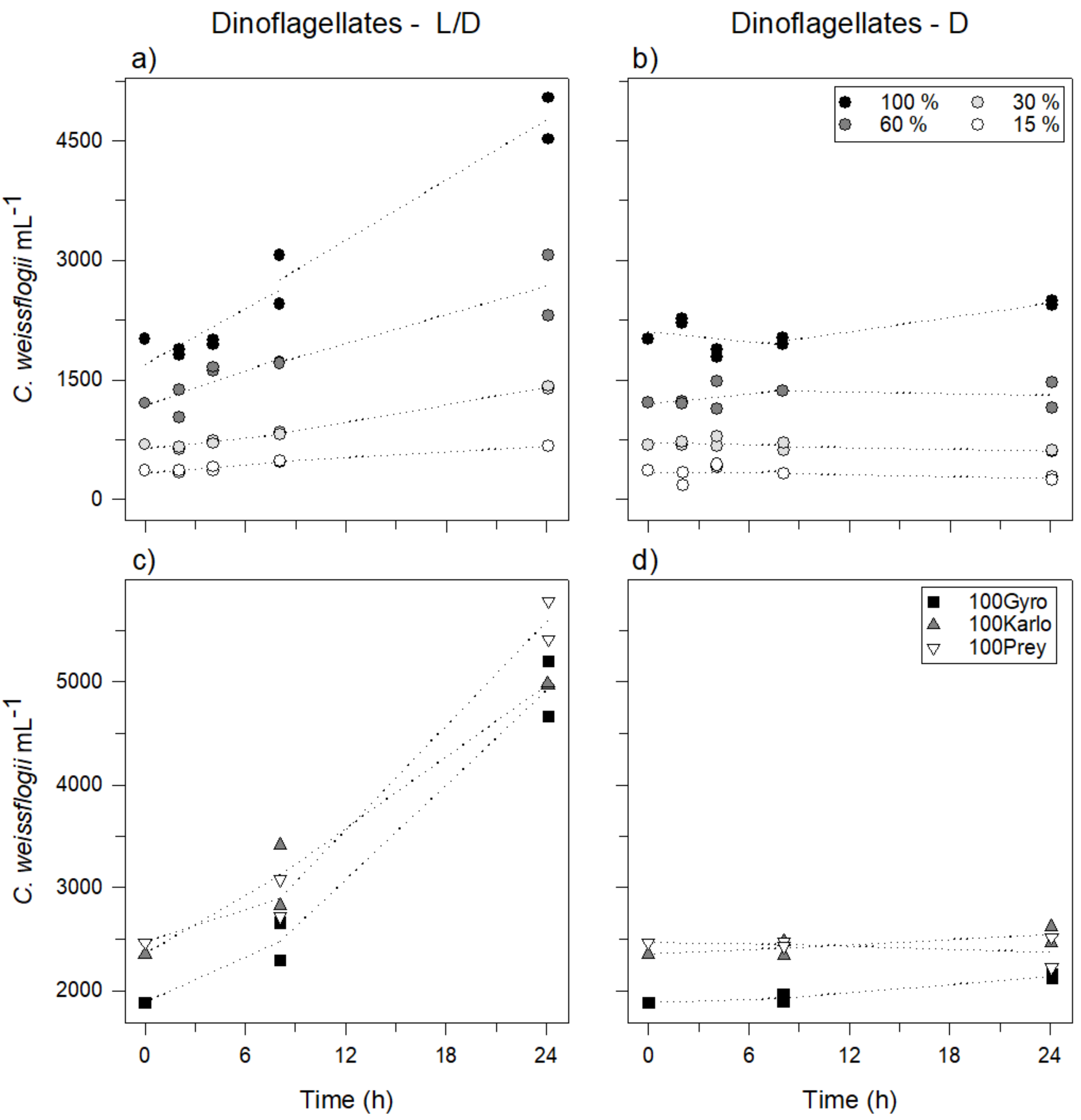

Figure S5. Abundance of *Contricriba weissflogii* over time for the experiment with dinoflagellates: a and b) dilution series in the L/D and D treatments respectively; c and d) control bottles series in the L/D and D treatments respectively.

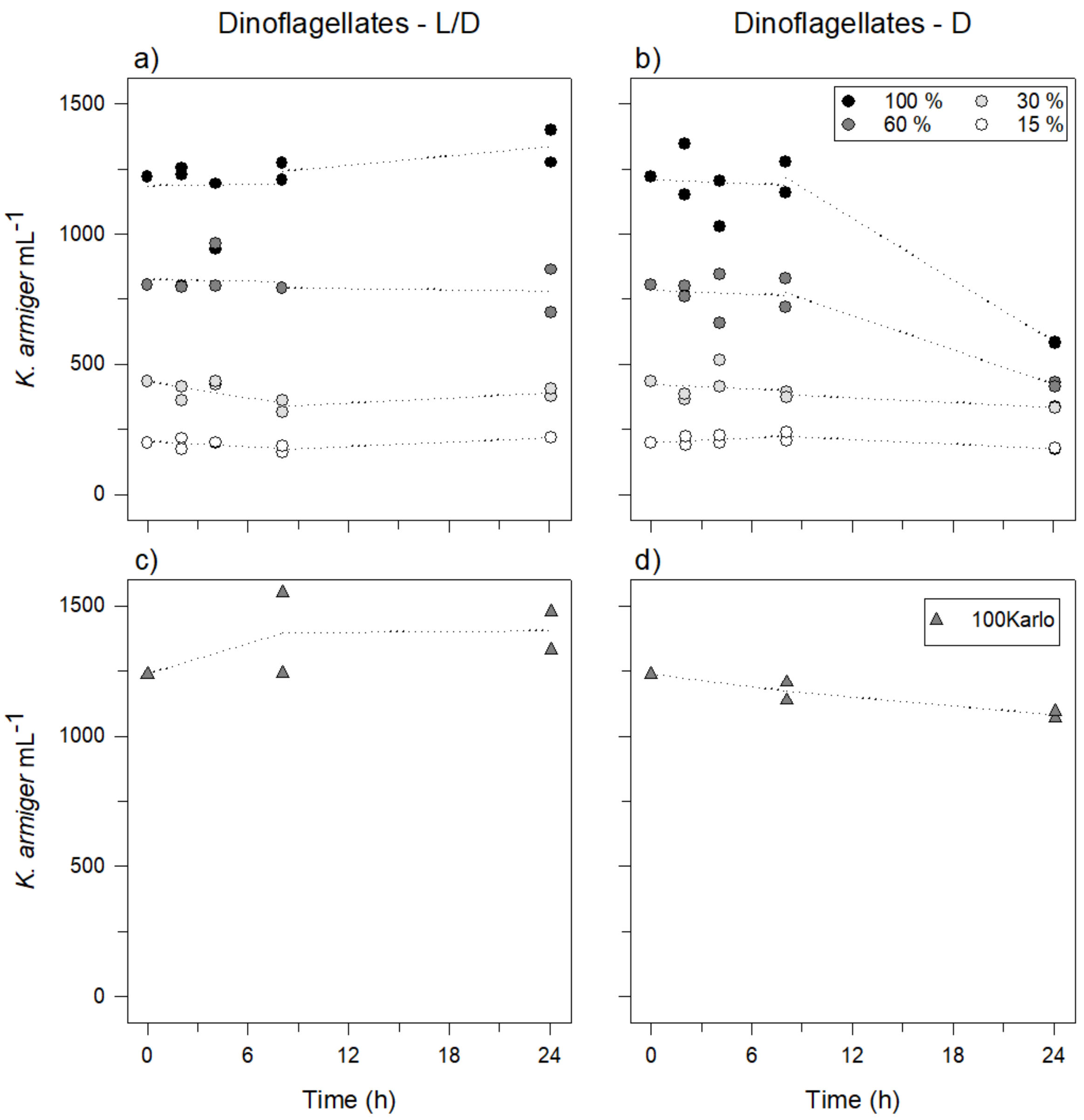

Figure S6. Abundance of *Karlodinium armiger* over time for the experiment with dinoflagellates: a and b) dilution series in the L/D and D treatments respectively; c and d) control bottles series in the L/D and D treatments respectively.

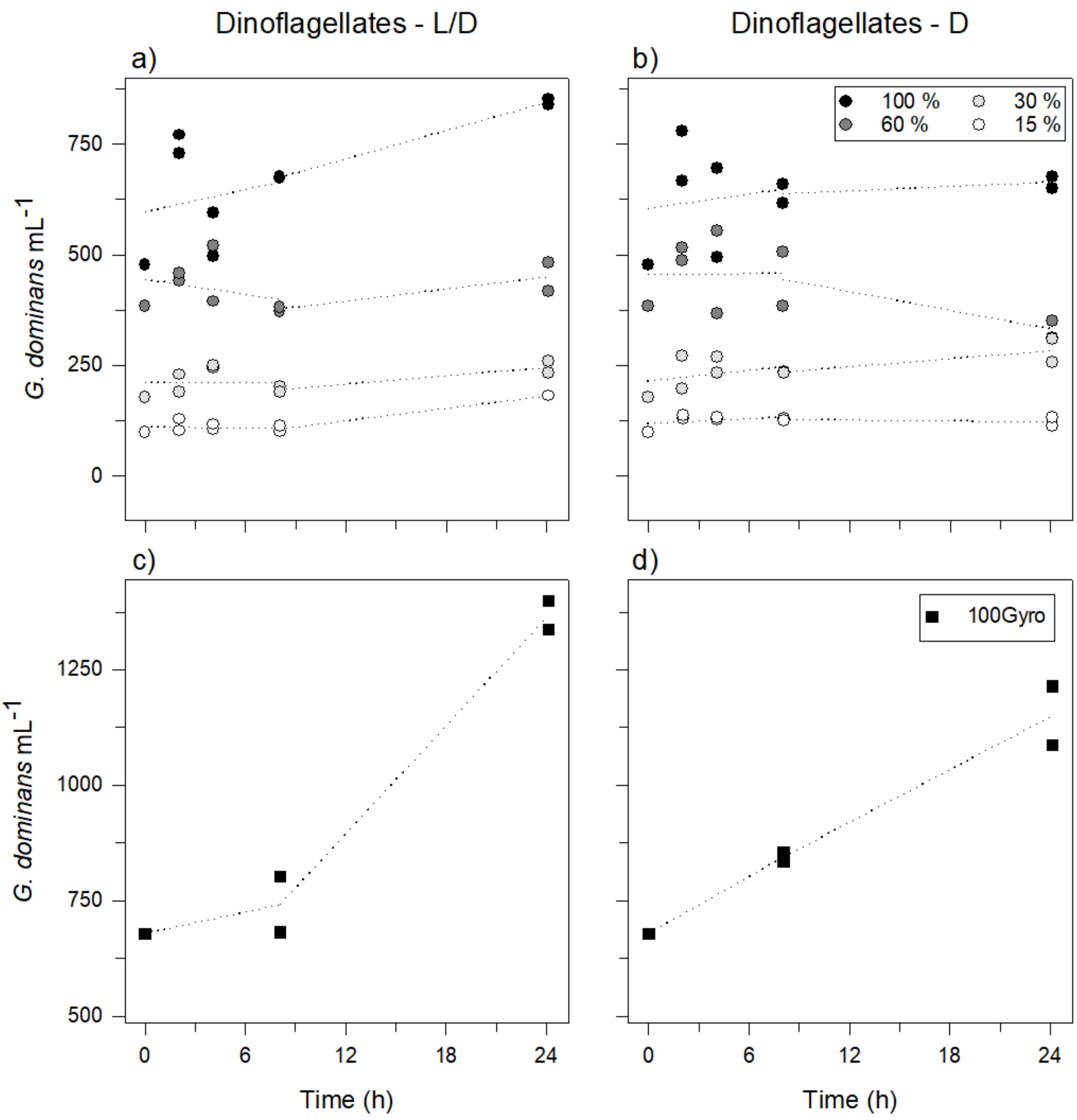

Figure S7. Abundance of *Gyrodinium dominans* over time for the experiment with dinoflagellates: a and b) dilution series in the L/D and D treatments respectively; c and d) control bottles series in the L/D and D treatments respectively.

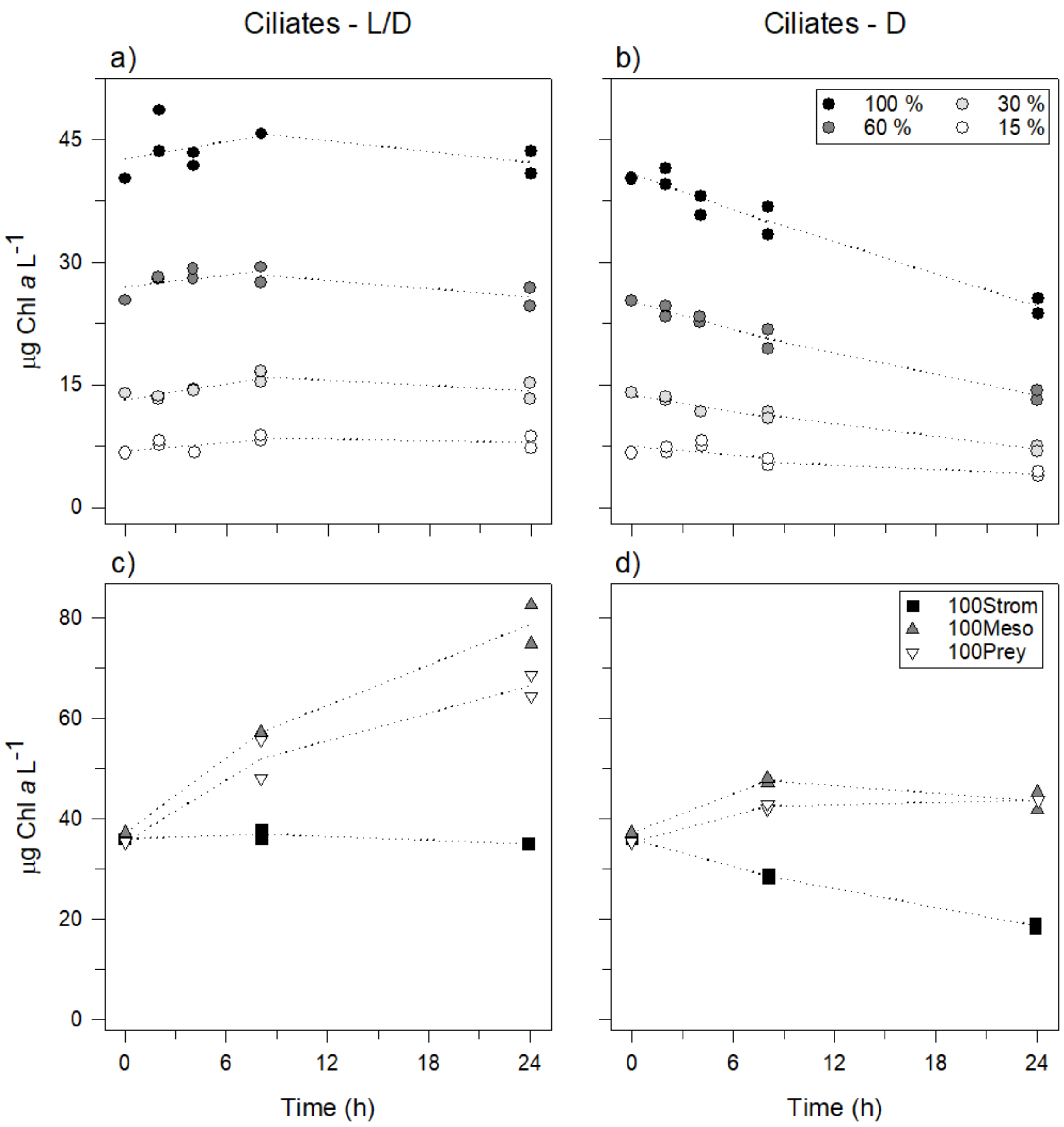

Figure S8. Chl *a* over time for the experiment with ciliates: a and b) dilution series in the L/D and D treatments respectively; c and d) control bottles series in the L/D and D treatments respectively.

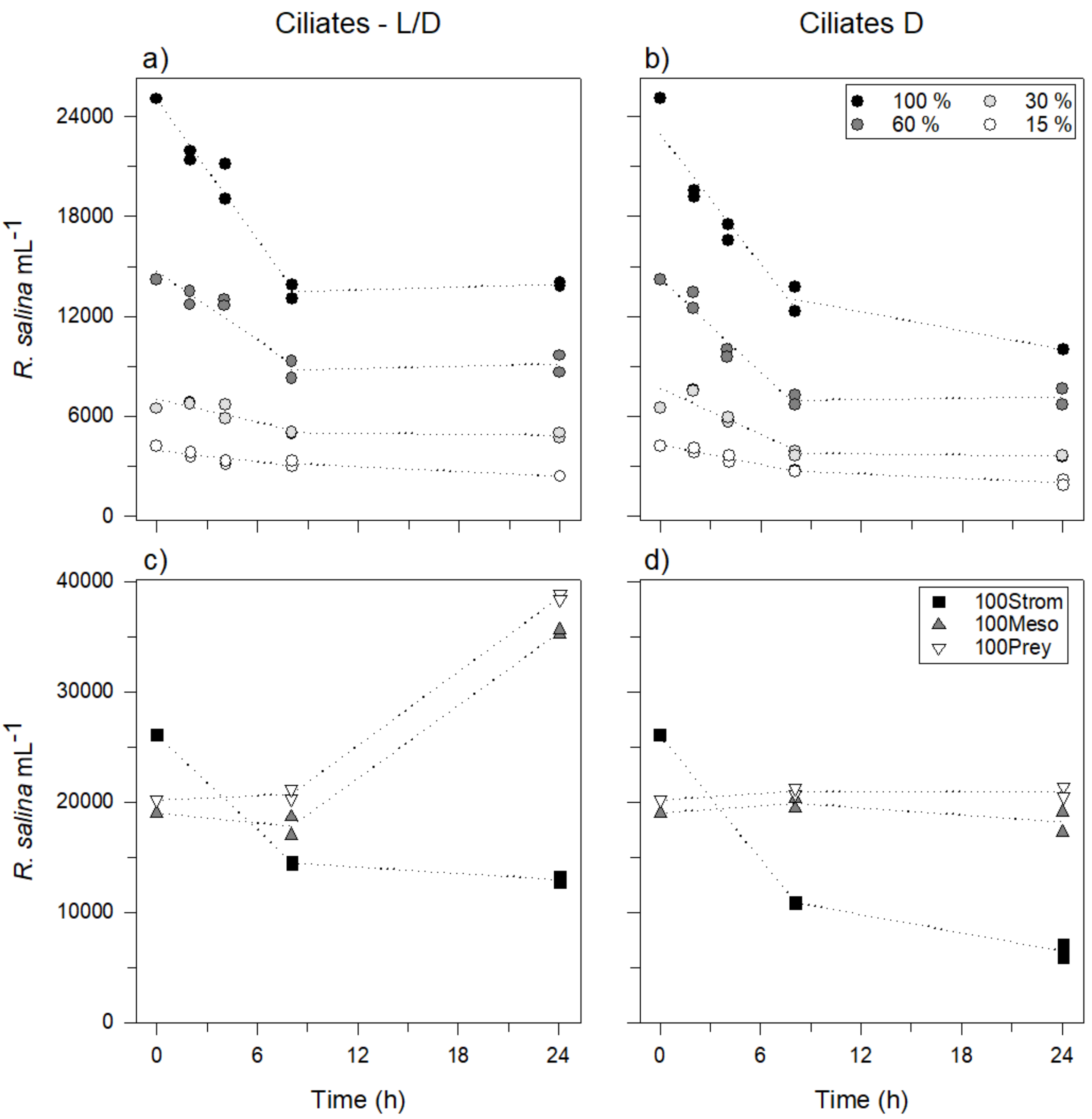

Figure S9. Abundance of *Rhodomonas salina* over time for the experiment with ciliates: a and b) dilution series in the L/D and D treatments respectively; c and d) control bottles series in the L/D and D treatments respectively.

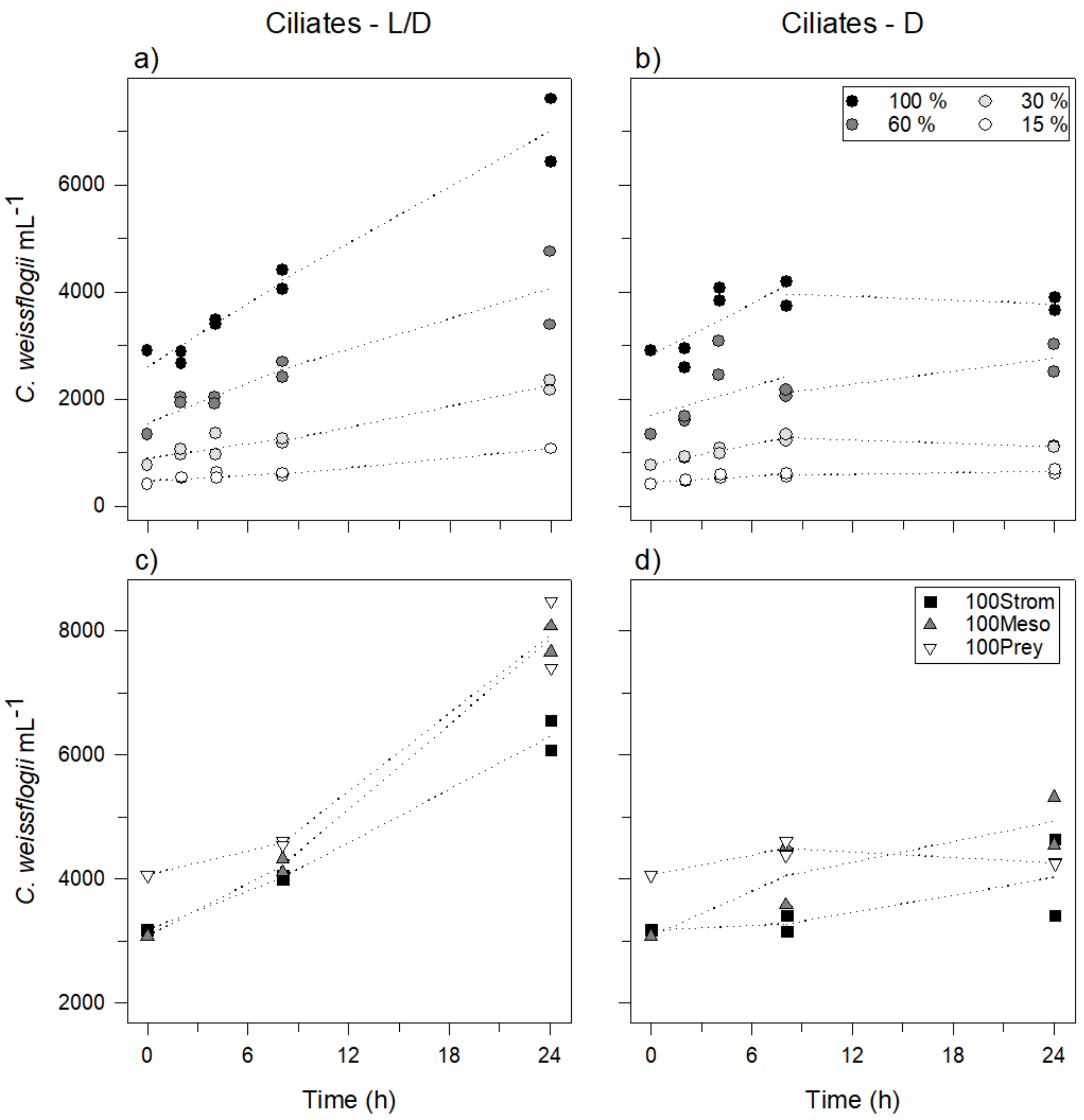

Figure S10. Abundance of *Conticribra weissflogii* over time for the experiment with ciliates: a and b) dilution series in the L/D and D treatments respectively; c and d) control bottles series in the L/D and D treatments respectively.

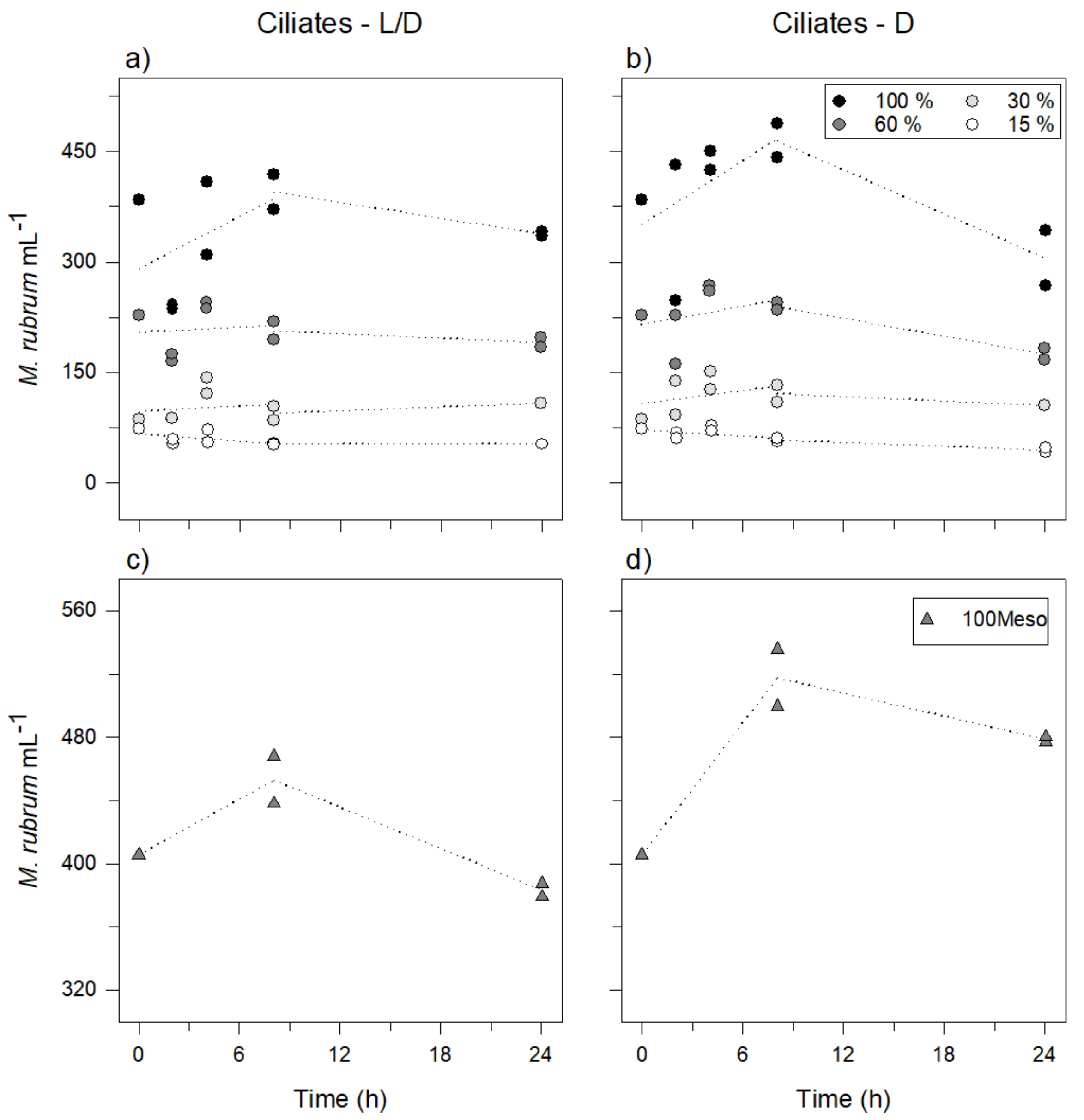

Figure S11. Abundance of *Mesodinium rubrum* over time for the experiment with ciliates: a and b) dilution series in the L/D and D treatments respectively; c and d) control bottles series in the L/D and D treatments respectively.

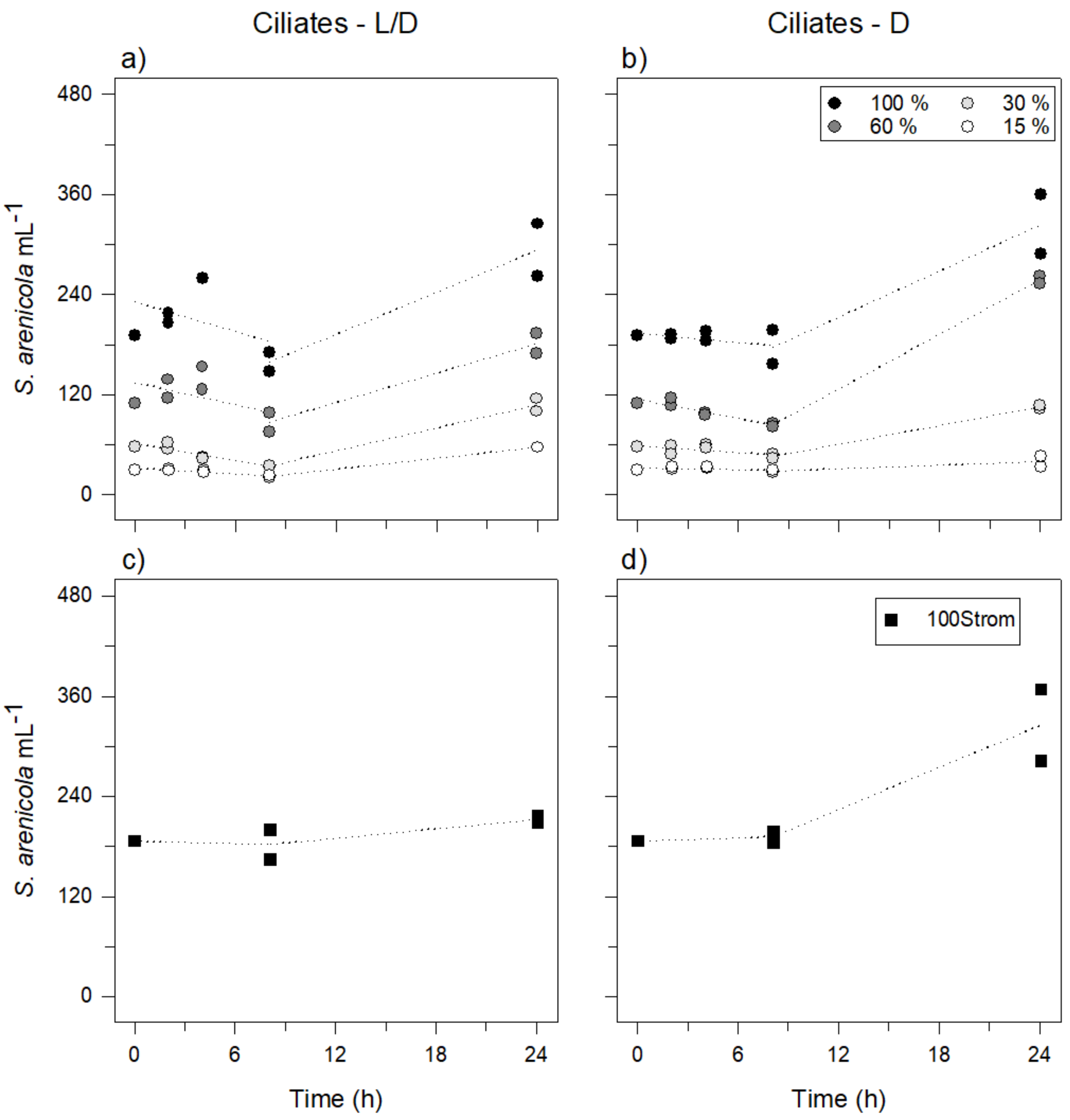

Figure S12. Abundance of *Strombidium arenicola* over time for the experiment with ciliates: a and b) dilution series in the L/D and D treatments respectively; c and d) control bottles series in the L/D and D treatments respectively.
